# miR-199a-3p Promotes Adipogenic Differentiation to Aggravate Steroid-Induced Osteonecrosis of Femoral Head via the ITGB8/FAK–ERK/RUNX2 Pathway

**DOI:** 10.34133/research.1186

**Published:** 2026-03-23

**Authors:** Wu Yang, Yunfei Yang, Mao Nie, Haobo Bai, Hongbo Zhang, Jian Zhang

**Affiliations:** ^1^ Department of Orthopedics, The First Affiliated Hospital of Chongqing Medical University, Chongqing 400016, China.; ^2^ Key Laboratory of Musculoskeletal Regeneration and Translational Medicine, Chongqing Municipal Health Commission, Chongqing 400016, China.; ^3^ Orthopaedic Research Laboratory of Chongqing Medical University, Chongqing 400016, China.; ^4^ Pharmaceutical Sciences Laboratory, Abo Akademi University, Turku 20520, Finland.; ^5^ Department of Geriatrics, The First Affiliated Hospital of Chongqing Medical University, Chongqing 400016, China.; ^6^ Department of Orthopedic Surgery, The Second Affiliated Hospital of Chongqing Medical University, Chongqing 400010, China.; ^7^ Turku Bioscience Centre, University of Turku and Åbo Akademi University, Turku 20520, Finland.

## Abstract

Steroid-induced osteonecrosis of the femoral head (SONFH) is a rapidly progressing and disabling complication of long-term glucocorticoid therapy, lacking effective early-stage intervention mechanisms. Its early manifestation involves a fate shift in bone marrow mesenchymal stem cells (BMSCs) characterized by decreased osteogenic differentiation (OGD) and increased adipogenic differentiation (AGD), yet the upstream regulatory mechanisms remain unclear. Herein, we integrated AGD-related microRNA (miRNA) microarray data with exosomal miRNA sequencing data and identified miR-199a-3p as a crucial candidate driver of this lineage imbalance. Our results revealed the up-regulation of miR-199a-3p in SONFH tissues and in glucocorticoid-treated cellular models, and indicated that its overexpression suppresses the OGD of BMSCs while markedly promoting the AGD. Further integrating mRNA-sequencing profiling during AGD with target prediction, protein–protein interaction network analysis, and dual-luciferase reporter assays, we confirmed integrin β8 (ITGB8) as a direct target of miR-199a-3p, which is consistently decreased in SONFH tissues and during adipogenic induction. We further revealed that miR-199a-3p suppressed the OGD of BMSCs by repressing ITGB8 expression, thereby inactivating the focal adhesion kinase (FAK)–extracellular signal-regulated kinase (ERK)–runt-related transcription factor 2 (RUNX2) signaling cascade. Conversely, silencing miR-199a-3p restores ITGB8 levels, reactivates this pathway, and corrects the OGD/AGD bias. In vivo, local administration of antagomiR-199a-3p in a SONFH rat model markedly improved trabecular bone architecture, increased bone mass, and up-regulated RUNX2 expression. These findings reveal for the first time that the miR-199a-3p/ITGB8–FAK–ERK–RUNX2 axis represents an unrecognized pathogenic pathway in SONFH, and support the local suppression of miR-199a-3p as a translatable early intervention strategy.

## Introduction

Steroid-induced osteonecrosis of the femoral head (SONFH) is triggered by prolonged exposure to glucocorticoids (GCs) [1,2]. Clinically, the cumulative incidence of osteonecrosis in corticosteroid-treated populations ranges from 9% to 40% [3]. Approximately 20,000 to 30,000 new cases emerge annually in the United States, accounting for roughly 10% of total hip replacements, highlighting its significant clinical burden [4]. Therefore, there is an urgent need to elucidate early pathogenic mechanisms and identify actionable targets. Emerging evidence suggests that the imbalanced differentiation of bone marrow mesenchymal stem cells (BMSCs) is a key event in the early stages of SONFH [5,6]. Specifically, GC exposure inhibits osteogenic differentiation (OGD) and promotes adipogenic differentiation (AGD), thereby driving the progression of bone structure degeneration and necrosis [7,8]. However, the upstream mechanisms driving OGD–AGD imbalance, particularly at the epigenetic level, remain unclear, limiting mechanistic elucidation and the development of therapeutic strategies.

Epigenetic regulation, especially microRNA (miRNA)-mediated post-transcriptional control, has been recognized as a key determinant of BMSC fate decisions and is increasingly implicated in GC-induced lineage imbalance [9,10]. Several miRNAs, including miR-601, miR-129-5p, and miR-130b-3p, have been shown to modulate OGD or AGD and contribute to SONFH-associated lineage dysregulation [11–13]. miR-199a-3p is a functional miRNA dysregulated within various bone metabolic disorders [14,15] and was markedly enriched in both bone tissues and exosomes derived from SONFH patients in our previous high-throughput screening [16], suggesting its potential role as a pathogenic regulator of lineage imbalance. However, whether miR-199a-3p actively participates in GC-related differentiation imbalance and exerts pathogenic effects through specific downstream signaling pathways remains unknown.

Integrins transmit extracellular matrix cues to intracellular signaling networks, thereby maintaining bone homeostasis and determining the fate of stem cells [17]. Several integrin subunits (e.g., ITGA5 and ITGB1) promote OGD by activating focal adhesion kinase (FAK) and its downstream extracellular signal-regulated kinase (ERK) pathway, thereby enhancing runt-related transcription factor 2 (RUNX2) transcriptional activity and driving OGD [18,19]. Integrin β8 (ITGB8) is also involved in the differentiation of MSCs [20]. Emerging evidence suggests that ITGB8 may participate in activating the FAK–ERK–RUNX2 axis, indicating a potentially essential role in OGD fate determination [21]. Furthermore, previous studies have demonstrated that miR-199a-3p can directly target and down-regulate the expression of ITGB8, providing crucial insights into the functional role of miR-199a-3p and its downstream mechanisms [22,23]. Nevertheless, whether the miR-199a-3p/ITGB8 axis functionally regulates the imbalance between OGD and AGD of BMSCs and whether it contributes to SONFH pathogenesis through the FAK–ERK pathway remains unclear.

**Fig. 1.**
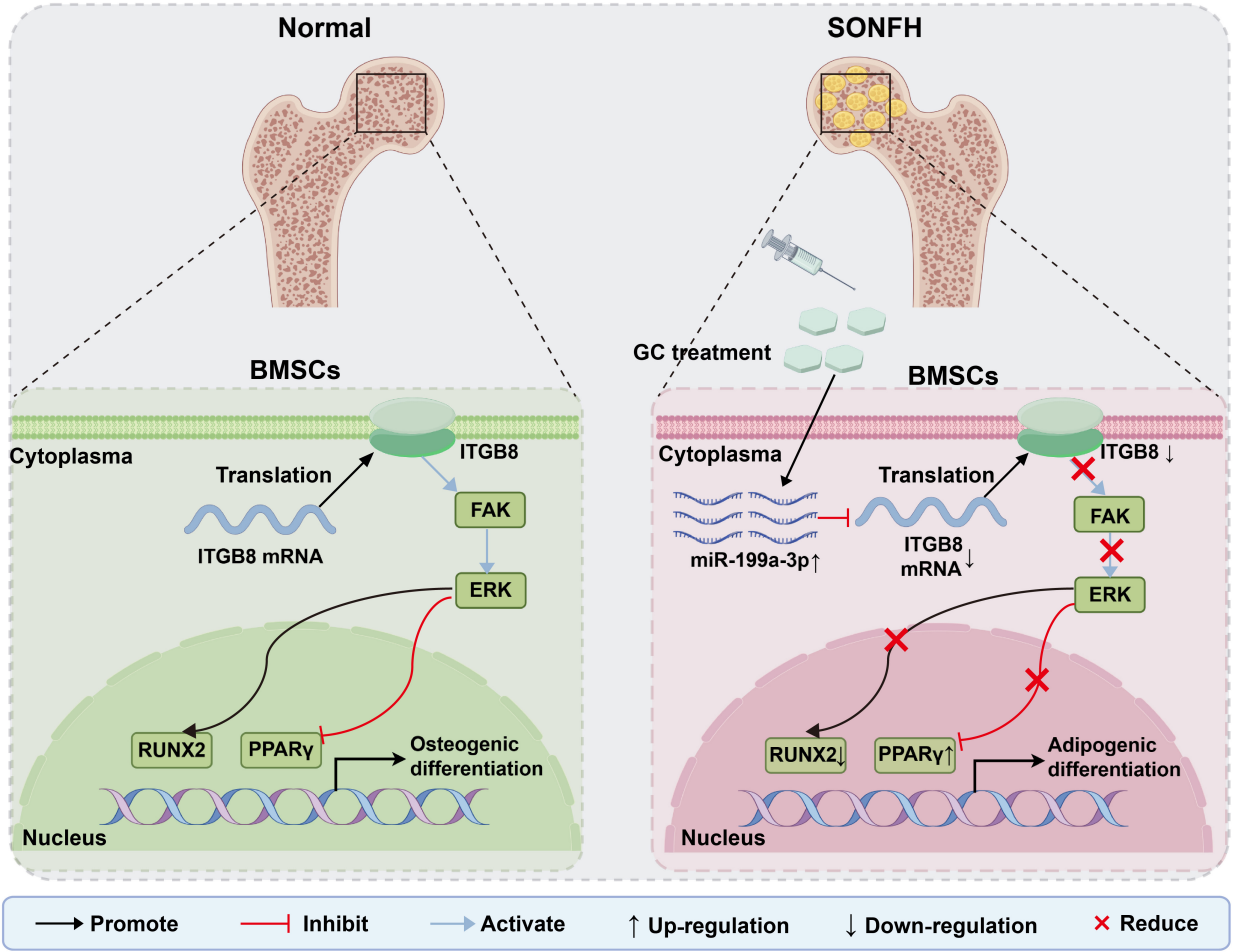
Schematic illustration of the miR-199a-3p/ITGB8/FAK–ERK–RUNX2 axis mediating the imbalance between osteogenic and adipogenic differentiation of BMSCs in SONFH.

Therefore, we hypothesize that GC exposure induces the up-regulation of miR-199a-3p, which subsequently targets ITGB8 to inhibit the FAK–ERK–RUNX2 osteogenic signaling pathway, thereby driving OGD–AGD imbalance in BMSCs and promoting SONFH progression; inhibiting miR-199a-3p may reverse these abnormalities (Fig. 1). Based on this hypothesis, this study aims to (a) integrate miRNA microarray data related to AGD of MSCs with exosomal miRNA sequencing profiles from SONFH patients to identify and clinically validate miR-199a-3p, (b) elucidate its regulatory role in OGD–AGD fate and the miR-199a-3p/ITGB8–FAK–ERK–RUNX2 mechanism in an in vitro GC model, and (c) evaluate the improvement of bone structure and differentiation phenotypes through local intervention targeting miR-199a-3p in animal models. Through these studies, we aim to establish a pathogenic regulatory framework centered on this axis, providing a basis for elucidating the early mechanisms of SONFH and developing interventionable targets.

## Results

### miR-199a-3p is increased in SONFH tissues and dexamethasone (DEX)-treated cells

**Fig. 2.**
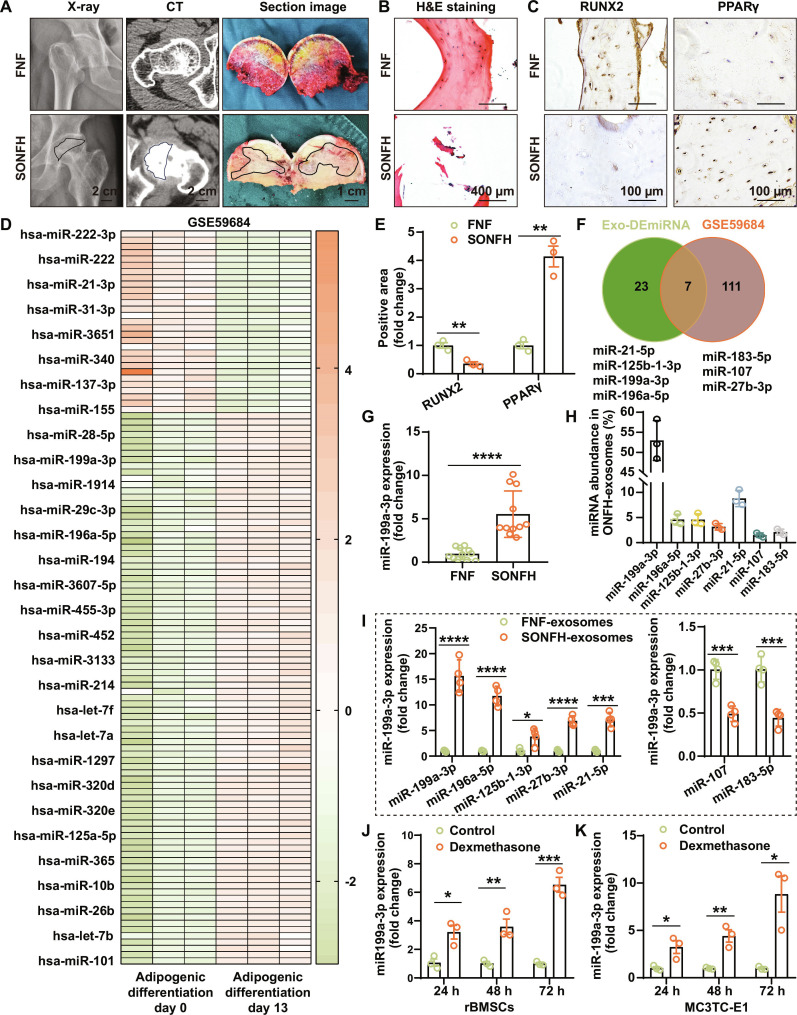
miR-199a-3p is increased in SONFH. (A) Representative radiographic, CT, and gross sectional images of femoral heads show femoral head collapse (circled areas indicate collapsed femoral head regions). (B) H&E staining of femoral head sections reveal bone necrosis and trabecular fractures. (C) IHC staining of bone tissues indicates down-regulation of RUNX2 and increased expression of PPARγ in SONFH. (D) Heatmap displaying 117 differentially expressed (DE)-miRNAs during AGD. (E) Quantitative analysis of RUNX2- and PPARγ-positive area (*n* = 3). (F) A Venn diagram for screening DE-miRNAs in SONFH exosomes with potential to be involved in AGD. (G) RT-qPCR analysis revealed that miR-199a-3p was up-regulated in SONFH samples (*n* = 11). (H) Abundance of 7 DE-miRNAs in SONFH exosomes shows the highest abundance of miR-199a-3p (*n* = 3). (I) RT-qPCR analysis of 7 DE-miRNAs in FNF exosomes and SONFH exosomes indicates that miR-199a-3p exhibits the highest fold up-regulation (*n* = 4). (J and K) RT-qPCR demonstrates sustained elevation of miR-199a-3p in dexamethasone-treated rBMSCs (J) and MC3T3-E1 cells (K) (both *n* = 3). [Data are presented as mean ± SD; Student’s *t* test or 2-way analysis of variance (ANOVA) was used; *P < 0.05, **P < 0.01, ***P < 0.001, ****P < 0.0001.]

We first collected and analyzed clinical specimens. Radiographic and histological examinations confirmed the typical pathological alterations of SONFH. Compared with femoral neck fracture (FNF) controls, x-ray and computed tomography (CT) imaging in SONFH patients revealed marked pathological changes (Fig. 2A). In addition, hematoxylin and eosin (H&E) staining showed disorganized trabecular architecture accompanied by extensive necrosis (Fig. 2B). Immunohistochemical (IHC) staining further demonstrated that RUNX2, a key transcription factor of OGD, was significantly down-regulated in SONFH tissues (only 35.6% of the FNF group; *P* < 0.01) (Fig. 2C and E). In contrast, the adipogenic regulator peroxisome proliferator-activated receptor γ (PPARγ) was markedly up-regulated (about 4.14-fold), indicating a disturbed balance between OGD and AGD.

Building on our previous work, which showed a pro-adipogenic effect of SONFH-derived exosomes [16], we next combined publicly available datasets to screen AGD-related miRNAs. Using the AGD dataset GSE59684 of MSCs, we compared expression profiles between day 0 and day 13 of adipogenic induction and identified 117 differentially expressed miRNAs, including 88 up-regulated and 27 down-regulated miRNAs (Fig. 2D and Table S1). Intersection of these miRNAs with 30 candidate miRNAs obtained from our prior exosomal sequencing yielded 7 potential key miRNAs (Fig. 2F). These key miRNAs include the up-regulated miR-27b-3p, miR-21-5p, miR-125b-1-3p, miR-199a-3p, and miR-196a-5p, along with the down-regulated miR-183-5p and miR-107 in SONFH exosomes.

The abundance of these 7 miRNAs in ONFH exosomes was further evaluated. As shown in Fig. 2H, miR-199a-3p accounted for approximately 53.0%, followed by miR-21-5p (8.8%), miR-196a-5p (4.6%), miR-125b-1-3p (4.5%), miR-27b-3p (3.2%), miR-183-5p (2.1%), and miR-107 (1.5%). We then examined their expression in FNF exosomes and SONFH exosomes. Real-time quantitative PCR (RT-qPCR) indicated that the level of miR-199a-3p in SONFH exosomes reached approximately 15.6-fold that of FNF exosomes, followed by miR-196a-5p (11.7-fold), miR-21-5p (7.1-fold), miR-27b-3p (6.9-fold), hsa-miR-125b-1-3p (3.8-fold), and the down-regulated miR-183-5p (0.44-fold) and miR-107 (0.49-fold) (all *P* < 0.05; Fig. 2I). This finding indicated miR-199a-3p as the most prominently altered miRNA in SONFH exosomes, suggesting that miR-199a-3p is most strongly associated with SONFH.

Given that miR-199a-3p exhibited the highest abundance (>50%) in ONFH exosomes and demonstrated the most significant alteration compared to FNF exosomes (up-regulation > 15-fold; *P* < 0.0001), it was selected for subsequent investigation. We further assessed its expression in bone tissues and cellular models. miR-199a-3p levels were markedly elevated in SONFH bone tissues, reaching approximately 5.5-fold of those in FNF samples (Fig. 2G). In vitro, treatment with 20 μM DEX for 72 h persistently induced miR-199a-3p expression in both rBMSCs and MC3T3-E1 cells (Fig. 2J and K).

Collectively, clinical specimen analyses, exosomal sequencing integration, and cell model validation consistently demonstrated up-regulation of miR-199a-3p during SONFH progression, indicating that it may act as a key upstream regulator of GC-induced OGD–AGD imbalance.

### miR-199a-3p inhibits OGD and promotes AGD

**Fig. 3.**
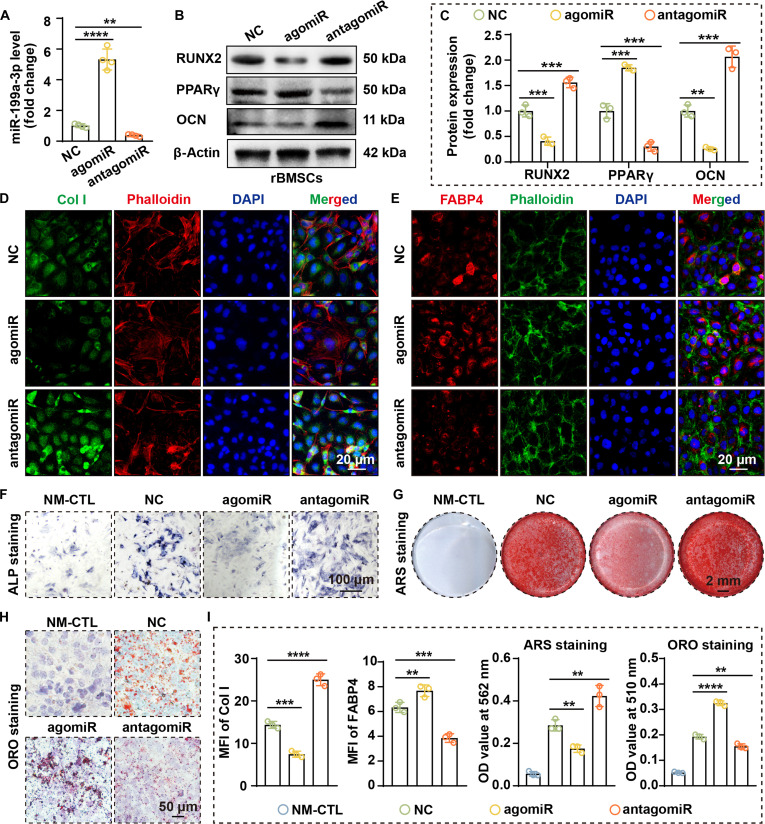
miR-199a-3p impairs OGD and promotes AGD in rBMSCs. (A) RT-qPCR shows that transfection with agomiR down-regulates miR-199a-3p, while transfection with antagomiR up-regulates miR-199a-3p (*n* = 4). (B) WB analysis indicates that miR-199a-3p overexpression down-regulates RUNX2 and OCN and up-regulates PPARγ protein levels. (C) Quantification of WB results (*n* = 3). (D and E) IF staining shows that miR-199a-3p overexpression down-regulates Col I (D) and up-regulates FABP4 (E). (F) ALP staining indicates that miR-199a-3p overexpression suppresses ALP expression (cells were cultured in OGD medium for 1 week). (G) Alizarin Red S (ARS) staining indicates that miR-199a-3p overexpression inhibits osteoblastic mineralization (cells were cultured in OGD medium for 3 weeks). (H) ORO staining indicates that miR-199a-3p overexpression promotes lipid accumulation (cells were cultured in AGD medium for 3 weeks). (I) Quantitative analysis of IF staining, ARS staining, and ORO staining (all *n* = 3). (Data are presented as mean ± SD; one-way ANOVA was used; *P < 0.05, **P < 0.01, ***P < 0.001, ****P < 0.0001.)

**Fig. 4.**
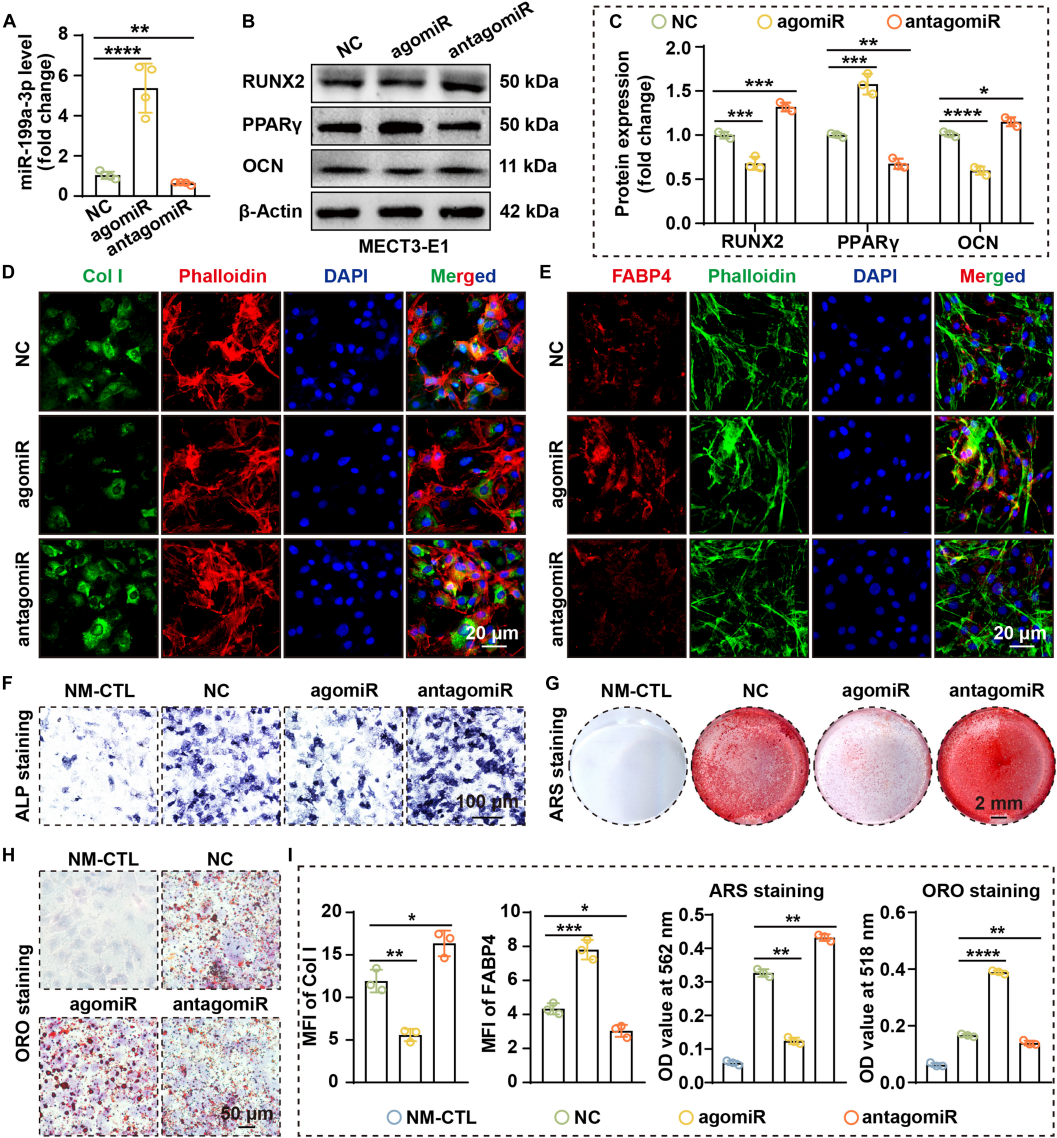
miR-199a-3p suppresses OGD and promotes AGD in MC3T3-E1 cells. (A) miR-199a-3p expression after transfection shows that transfection with agomiR down-regulates miR-199a-3p, while transfection with antagomiR up-regulates miR-199a-3p (*n* = 4). (B) WB analysis indicates that miR-199a-3p overexpression down-regulates RUNX2 and OCN and up-regulates PPARγ protein levels. (C) Quantification of WB results (*n* = 3). (D and E) IF staining shows that miR-199a-3p overexpression down-regulates Col I (D) and up-regulates FABP4 (E). (F) ALP staining indicates that miR-199a-3p overexpression suppresses ALP expression (cells were cultured in OGD medium for 1 week). (G) ARS staining indicates that miR-199a-3p overexpression inhibits osteoblastic mineralization (cells were cultured in OGD medium for 3 weeks). (H) ORO staining indicates that miR-199a-3p overexpression promotes lipid accumulation (cells were cultured in AGD medium for 3 weeks). (I) Quantitative analysis of IF staining, ARS staining, and ORO staining (all *n* = 3). (Data are presented as mean ± SD; one-way ANOVA was used; *P < 0.05, **P < 0.01, ***P < 0.001, ****P < 0.0001.)

To clarify the function of miR-199a-3p in OGD–AGD, we established gain- and loss-of-function models in the 2 cells. RT-qPCR confirmed that agomiR-199a-3p significantly enhanced miR-199a-3p expression (4.3-fold in rBMSCs and 5.4-fold in MC3T3-E1 cells; both *P* < 0.0001), whereas antagomiR-199a-3p effectively reduced its expression (by 61.3% in rBMSCs and 63.7% in MC3T3-E1 cells) (Figs. 3A and 4A). Additionally, RT-qPCR results demonstrated that agomiR-199a-3p down-regulated RUNX2 and OCN expression while simultaneously up-regulating PPARγ and FABP4 expression (Figs. S1 and S2).

Western blotting (WB) analysis showed that agomiR-199a-3p down-regulated RUNX2 and osteocalcin (OCN) while up-regulating PPARγ (Figs. 3B and C and 4B and C). Immunofluorescence (IF) staining further confirmed that agomiR-199a-3p reduced expression of the OGD marker type I collagen (Col I) and enhanced the adipogenic marker FABP4 (Figs. 3D, E, and I and 4D, E, and I). In contrast, miR-199a-3p silencing increased the expression of OGD-related markers and suppressed the expression of adipogenic markers.

At the functional level, miR-199a-3p overexpression decreased alkaline phosphatase (ALP) activity (Figs. 3F and 4F) and reduced mineralized nodule formation (Figs. 3G and I and 4G and I) while promoting lipid accumulation (Figs. 3H and I and 4H and I). Conversely, miR-199a-3p inhibition effectively restored osteogenic phenotypes and reduced lipid droplet formation. These results indicate that miR-199a-3p exerts bidirectional regulation of differentiation, suppressing OGD and enhancing AGD, and may be crucial in SONFH pathogenesis.

### ITGB8 is a direct target of miR-199a-3p

**Fig. 5.**
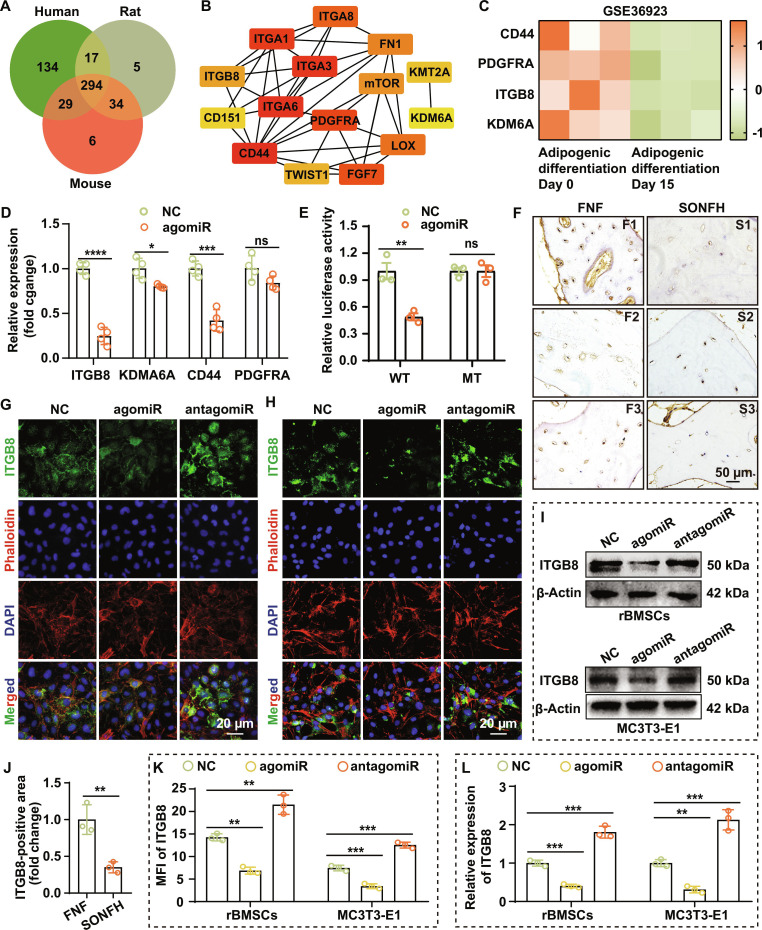
ITGB8 is a direct target of miR-199a-3p. (A) Venn diagram showing the predicted target genes of miR-199a-3p conserved across human, rat, and mouse. (B) Top 15 hub genes among the conservatively predicted targets of miR-199a-3p. (C) Heatmap illustrating the expression levels of CD44, KDM6A, ITGB8, and PDGFRA during AGD. (D) RT-qPCR analysis of CD44, KDM6A, ITGB8, and PDGFRA in rBMSCs transfected with agomiR-199a-3p shows the most significant down-regulation of ITGB8 (*n* = 4). (E) Dual-luciferase reporter assay indicates that ITGB8 is a direct target gene of miR-199a-3p (*n* = 3). (F) IHC staining reveals down-regulation of ITGB8 in SONFH bone tissue. (G and H) IF staining indicates that overexpression of miR-199a-3p suppresses ITGB8 expression in rBMSCs (G) and MC3T3-E1 cells (H). (I) WB analysis reveals that overexpression of miR-199a-3p suppresses ITGB8 expression. (J to L) Quantification of ITGB8 expression based on IHC staining (J), IF staining (K), and WB (L) results (all *n* = 3). (Data are presented as mean ± SD; one-way ANOVA was used; ns, *P* > 0.05, *P < 0.05, **P < 0.01, ***P < 0.001, ****P < 0.0001.)

To elucidate the mechanism by which miR-199a-3p regulates differentiation, we first used TargetScan to predict its potential targets. The analysis identified 474, 350, and 363 conserved target genes in human, rat, and mouse, respectively, with 294 genes shared across all 3 species (Table S2 and Fig. 5A). These 294 genes were subjected to protein–protein interaction (PPI) analysis to construct a PPI network (Fig. S3), and 15 hub genes were thereby identified, including CD44, ITGA6, ITGA3, ITGA1, PDGFRA, FGF7, ITGA8, LOX, FN1, MTOR, ITGB8, TWIST1, KMT2A, CD151, and KDM6A (Fig. 5B).

To further pinpoint target genes involved in adipogenic regulation, we intersected these hub genes with differentially expressed genes (DEGs) from the AGD dataset GSE36923. This dataset yielded 4,084 DEGs, including 1,956 up-regulated and 2,128 down-regulated genes (Table S3). Cross-analysis identified 4 miR-199a-3p candidate targets that were altered during AGD: CD44, KDM6A, ITGB8, and PDGFRA (Fig. 5C and Fig. S4).

We then validated the responsiveness of these 4 candidates to miR-199a-3p in rBMSCs. RT-qPCR showed that agomiR-199a-3p markedly decreased ITGB8 (to 24.7% of control), CD44 (42.2%), and KDM6A (80.2%) expression (Fig. 5D). In contrast, PDGFRA showed a downward trend without reaching statistical significance (Fig. 5D). Among them, ITGB8 exhibited the greatest change, suggesting it is the most likely functional target.

Sequence alignment revealed 2 highly conserved miR-199a-3p binding sites within the 3′ untranslated region (UTR) of ITGB8 (Fig. S5). Dual-luciferase reporter assays further showed that miR-199a-3p suppressed luciferase activity driven by the wild-type (WT)-ITGB8 3′UTR, but had no appreciable effect on the corresponding mutant (MT) constructs (Fig. 5E), confirming this specific interaction.

At the cellular level, miR-199a-3p overexpression markedly decreased ITGB8 protein in both rBMSCs and MC3T3-E1 cells (Fig. 5G to I, K, and L). Consistently, IHC analysis of clinical samples showed significantly reduced ITGB8 expression in femoral head tissues from SONFH patients (*P* < 0.01; Fig. 5F and J), in line with the in vitro findings. Together, these results demonstrate that miR-199a-3p directly targets ITGB8, and that ITGB8 down-regulation is likely involved in the pathological process of SONFH.

### miR-199a-3p inactivates the FAK–ERK–RUNX2 pathway via ITGB8 to block OGD

**Fig. 6.**
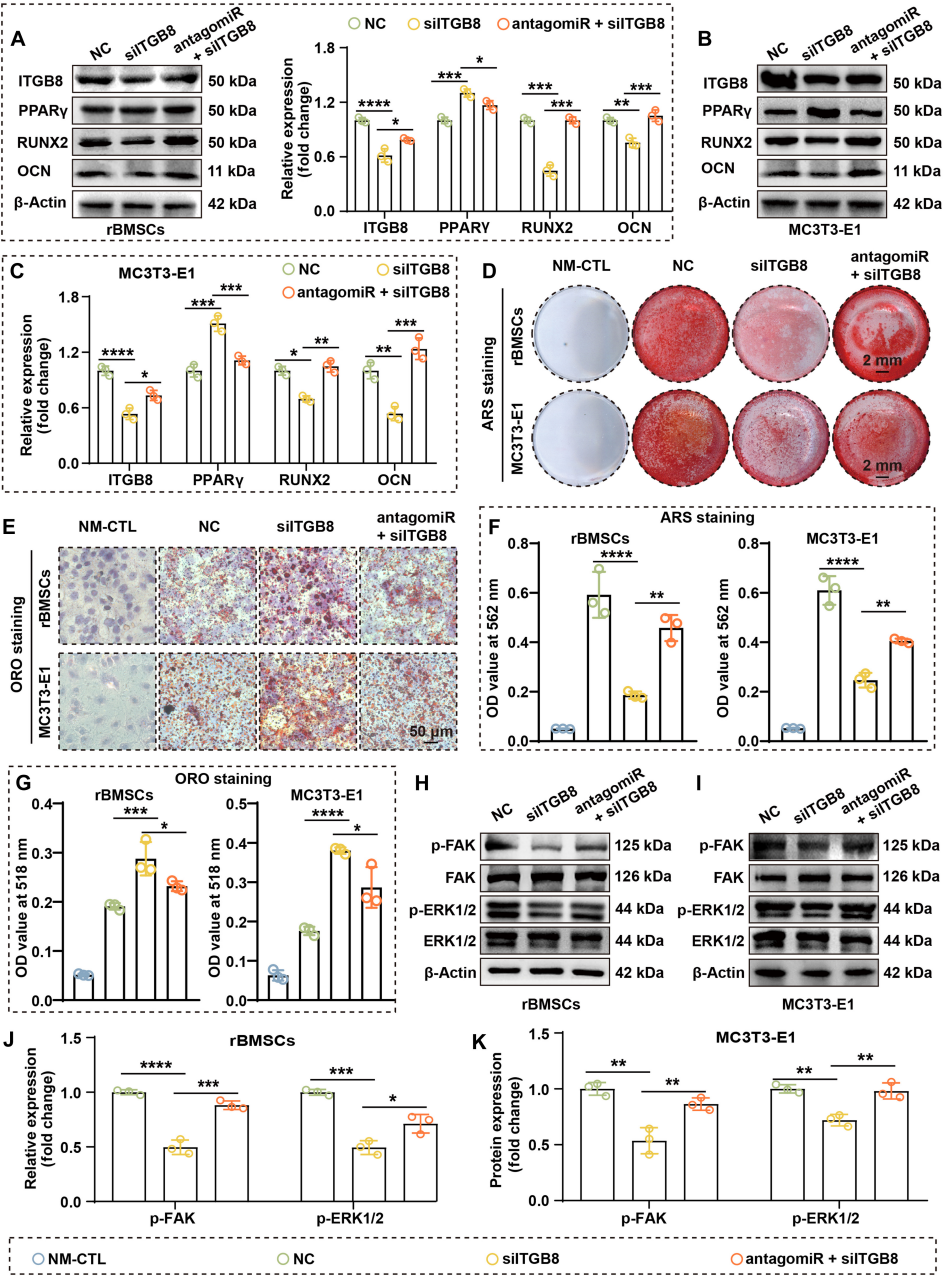
MiR-199a-3p inhibits OGD through the ITGB8–FAK–ERK–RUNX2 axis. (A and B) WB analysis demonstrates that small interfering RNA (siRNA)-mediated knockdown of ITGB8 down-regulates RUNX2 and OCN and up-regulates PPARγ, while simultaneous silencing of miR-199a-3p rescues this effect (*n* = 3). (C) Quantification of WB results in MC3T3-E1 cells (*n* = 3). (D) ARS staining reveals that ITGB8 knockdown inhibits osteogenic mineralization, while simultaneous silencing of miR-199a-3p enhances osteogenic mineralization. (E) ORO staining indicates that ITGB8 knockdown promotes lipid accumulation, while simultaneous silencing of miR-199a-3p reduces this promotion. (F and G) Quantitative analysis of ARS staining (cells were cultured in OGD medium for 3 weeks) (F) and ORO staining (cells were cultured in AGD medium for 3 weeks) (G) (both *n* = 3). (H and I) WB analysis of FAK, p-FAK, ERK1/2, and p-ERK1/2 shows that ITGB8 knockdown inhibits the phosphorylation levels of FAK and ERK1/2 in rBMSCs (H) and MC3T3-E1 cells (I), while simultaneous silencing of miR-199a-3p alleviates this inhibitory effect. (J and K) Quantification of WB results (both *n* = 3). (Data are presented as mean ± SD; one-way ANOVA was used; *P < 0.05, **P < 0.01, ***P < 0.001, ****P < 0.0001.)

Given that ITGB8 was identified as a direct target of miR-199a-3p, we next investigated its functional role in regulating the balance between OGD and AGD. ITGB8 expression was silenced using siITGB8, resulting in a reduction of ITGB8 protein levels to 61.5% and 53.4% of control, respectively (Fig. 6A to C). Additionally, RT-qPCR results demonstrated that siITGB8 down-regulated RUNX2 and OCN expression while simultaneously up-regulating PPARγ and FABP4 expression (Figs. S6 and S7). Functionally, ITGB8 knockdown impaired OGD, as evidenced by the down-regulation of RUNX2 and OCN and decreased mineralized nodule formation (Fig. 6A to D and F). In addition, lipid droplet accumulation and expression of the adipogenic marker PPARγ were increased, indicating enhanced AGD (Fig. 6E and G).

To determine whether these effects were mediated by miR-199a-3p, we performed rescue experiments using antagomiR-199a-3p. Cotransfection with antagomiR-199a-3p restored ITGB8 expression to 128.1% (rBMSCs) and 137.3% (MC3T3-E1) of the siITGB8 group, partially reversing the suppression of OGD and promotion of AGD induced by ITGB8 knockdown (Fig. 6A to C). Additionally, RT-qPCR results demonstrated that cotransfection with antagomiR-199a-3p promoted the expression of RUNX2 and OCN and reduced the expression of PPARγ and FABP4 (Figs. S6 and S7). These findings suggest that ITGB8 serves as a crucial mediator of miR-199a-3p-driven regulation of differentiation imbalance.

Based on Kyoto Encyclopedia of Genes and Genomes (KEGG) pathway analysis and previous reports, we hypothesized that this process involves the downstream FAK–ERK–RUNX2 signaling axis of ITGB8. WB demonstrated that ITGB8 knockdown markedly reduced p-FAK, p-ERK1/2, and RUNX2, whereas simultaneous silencing of miR-199a-3p partially restored this pathway (Fig. 6H to K). These findings indicate that miR-199a-3p inactivates the FAK–ERK–RUNX2 cascade by targeting ITGB8, thereby blocking the OGD of BMSCs and driving their shift toward an adipogenic lineage.

### Silencing miR-199a-3p attenuates dexamethasone-induced impairment of cell differentiation

Given the high expression of miR-199a-3p in SONFH and its role in mediating differentiation imbalance, we further explored its therapeutic potential in a GC-induced cell injury model. Treatment with 20 μM DEX markedly induced miR-199a-3p expression, whereas miR-199a-3p knockdown reduced its levels to 35.1% (rBMSCs) and 44.8% (MC3T3-E1) of those in the DEX + NC group (Fig. S8A and B).

**Fig. 7.**
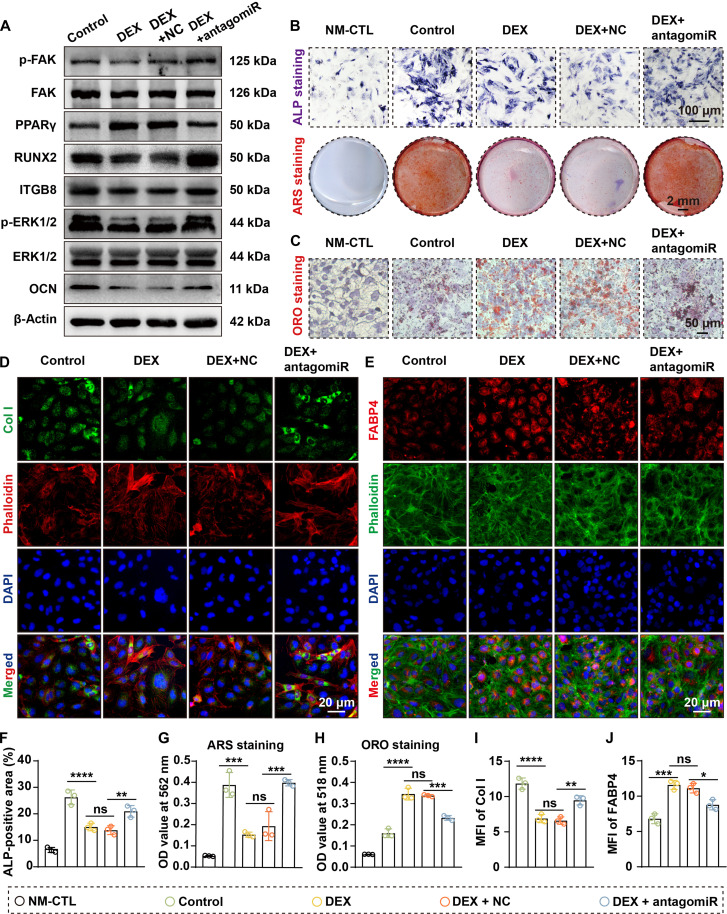
Silencing miR-199a-3p rescues DEX-induced impairment of OGD in rBMSCs. (A) WB analysis of key signaling molecules and differentiation markers. Results indicate that DEX inhibits the levels of ITGB8, RUNX2, OCN, p-ERK1/2, and p-FAK while elevating PPARγ levels, while simultaneous silencing of miR-199a-3p rescues these alterations. (B) ALP staining (cells were cultured in OGD medium for 1 week) and ARS staining (cells were cultured in OGD medium for 3 weeks) reveal that DEX suppressed ALP expression and osteogenic mineralization, while simultaneous miR-199a-3p silencing alleviated this inhibition. (C) ORO staining shows that DEX promotes lipid accumulation in rBMSCs, while simultaneous silencing of miR-199a-3p alleviates this promotion (cells were cultured in AGD medium for 3 weeks). (D and E) IF staining of Col I (D) and FABP4 (E) indicates that DEX down-regulated Col I and up-regulated FABP4, while simultaneous silencing of miR-199a-3p alleviated these effects. (F to H) Quantitative analysis of ALP staining (F), ARS staining (G), and ORO staining (H) (all *n* = 3). (I and J) Quantification of Col I (I) and FABP4 (J) IF staining (both *n* = 3). (Data are presented as mean ± SD; one-way ANOVA was used; ns, *P* > 0.05, *P < 0.05, **P < 0.01, ***P < 0.001, ****P < 0.0001.)

**Fig. 8.**
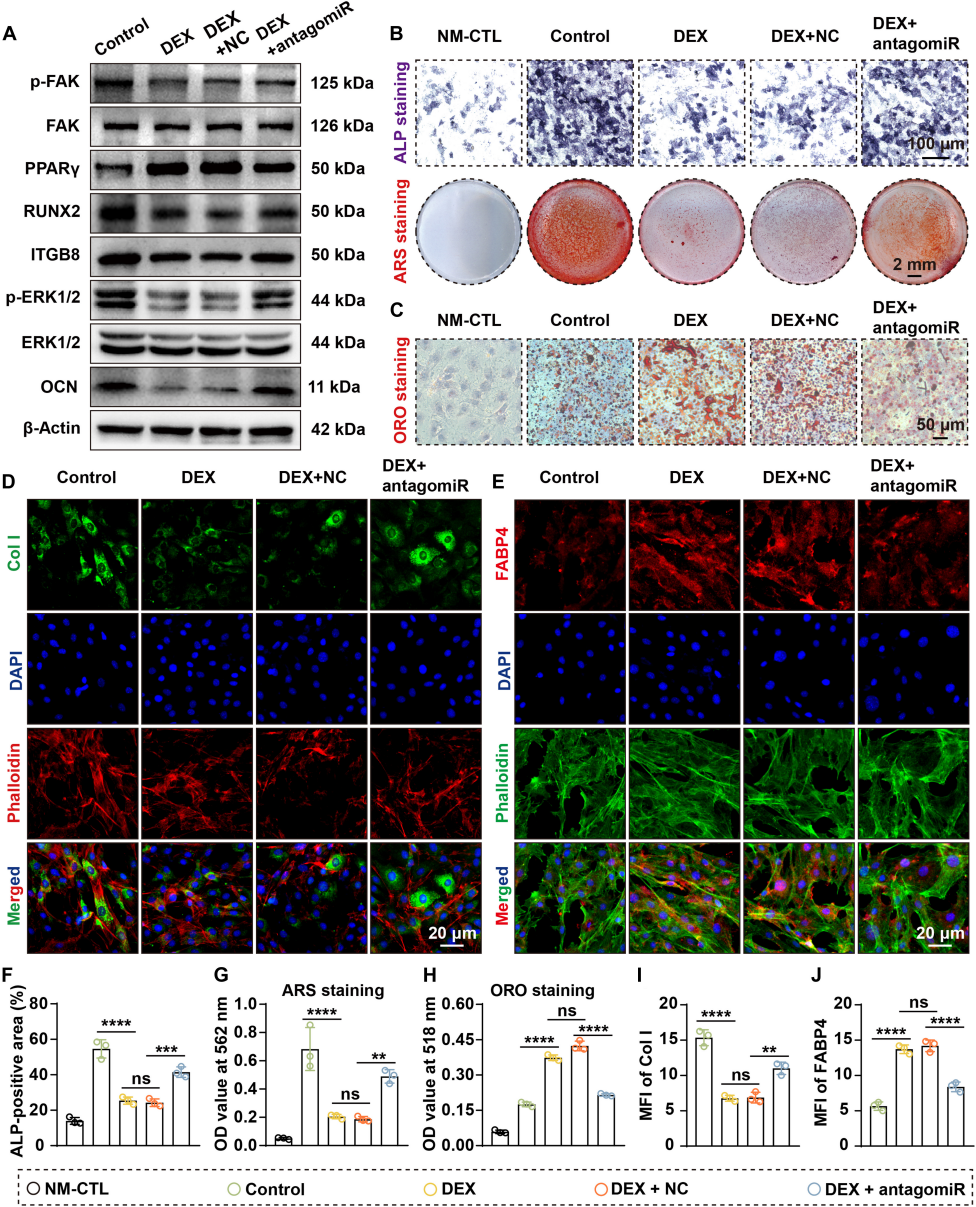
Silencing miR-199a-3p alleviates DEX-induced impairment of OGD in MC3T3-E1 cells. (A) WB analysis of signaling molecules and differentiation markers. Results indicate that DEX inhibits the levels of ITGB8, RUNX2, OCN, p-ERK1/2, and p-FAK while elevating PPARγ levels, while simultaneous silencing of miR-199a-3p rescues these alterations. (B) ALP staining (cells were cultured in OGD medium for 1 week) and ARS staining (cells were cultured in OGD medium for 3 weeks) reveal that DEX suppressed ALP expression and osteogenic mineralization, while simultaneous miR-199a-3p silencing alleviated this inhibition. (C) ORO staining shows that DEX promotes lipid accumulation in MC3T3-E1 cells, while simultaneous silencing of miR-199a-3p alleviates this promotion (cells were cultured in AGD medium for 3 weeks). (D and E) IF staining of Col I (D) and FABP4 (E) indicates that DEX down-regulated Col I and up-regulated FABP4, while simultaneous silencing of miR-199a-3p alleviated these effects. (F to H) Quantitative analysis of ALP staining (F), ARS staining (G), and ORO staining (H) (all *n* = 3). (I and J) Quantification of Col I (I) and FABP4 (J) IF staining (both *n* = 3). (Data are presented as mean ± SD; one-way ANOVA was used; ns, *P* > 0.05, *P < 0.05, **P < 0.01, ***P < 0.001, ****P < 0.0001.)

At the phenotypic level, RT-qPCR showed that DEX treatment down-regulated the expression of osteogenic markers (RUNX2 and OCN) and up-regulated the adipogenic markers (FABP4 and PPARγ) (Figs. S9 and S10). Silencing miR-199a-3p partially reversed this change. Additionally, WB analysis revealed that DEX treatment down-regulated the osteogenic proteins RUNX2 and OCN and up-regulated the adipogenic factor PPARγ. Silencing miR-199a-3p partially restored the expression of RUNX2 and OCN and suppressed the DEX-induced increase in PPARγ (Figs. 7A and 8A). IF staining further confirmed that antagomiR-199a-3p reversed the DEX-induced reduction in Col I expression and the increase in FABP4 levels (Figs. 7D, E, I, and J and 8D, E, I, and J).

To further clarify whether these effects were mediated through the miR-199a-3p/ITGB8 axis, we examined the associated downstream signaling pathways. WB revealed that DEX treatment suppressed ITGB8 expression and reduced p-FAK and p-ERK1/2 levels, whereas miR-199a-3p silencing markedly restored ITGB8 expression and reactivated p-FAK and p-ERK1/2 (Figs. 7A and 8A).

Functionally, DEX treatment significantly decreased ALP activity in rBMSCs (by 42.7%), inhibited mineralized nodule formation (by 60.3%), and markedly increased lipid droplet accumulation (by 1.6-fold) (all *P* < 0.001; Fig. 7B, C, and F to H). These deleterious effects were substantially alleviated by the knockdown of miR-199a-3p. Same trends were observed in MC3T3-E1 (Fig. 8B, C, and F to H). Collectively, silencing miR-199a-3p restores ITGB8–FAK–ERK signaling activity and mitigates GC-induced suppression of OGD and enhancement of AGD, underscoring its potential therapeutic value.

### Local silencing of miR-199a-3p improves femoral head microarchitecture and bone formation in rats

Given that in vitro experiments demonstrated that miR-199a-3p silencing effectively alleviates GC-induced OGD–AGD imbalance, we next evaluated its therapeutic potential in vivo using a rat SONFH model. SONFH was induced by methylprednisolone (MPS) administration, followed by local injection of antagomiR-199a-3p.

**Fig. 9.**
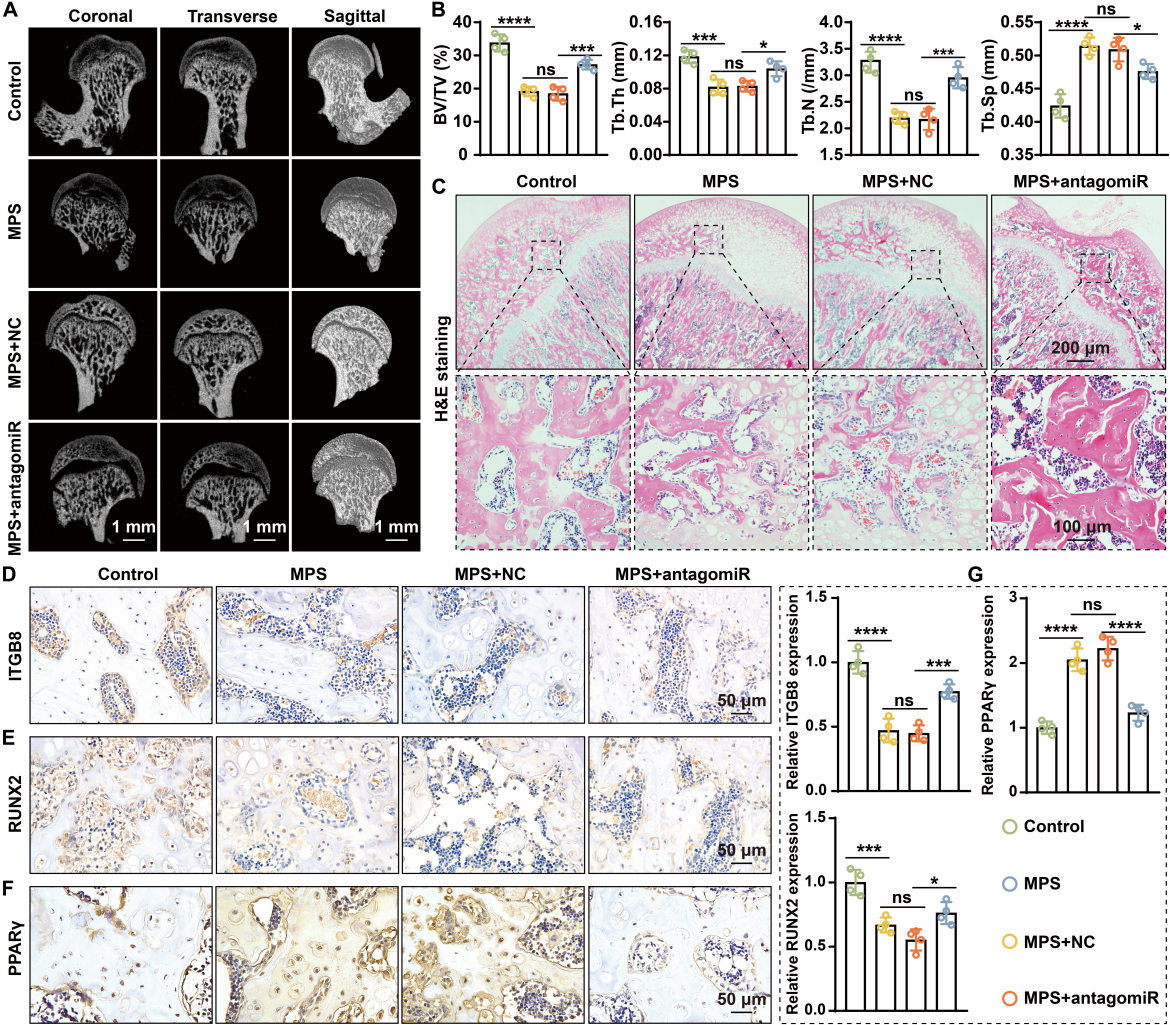
Silencing miR-199a-3p protects against GC-induced SONFH in rats. (A) Representative micro-CT images in coronal, transverse, and sagittal planes reveal that GC treatment induces femoral head necrosis, while simultaneous silencing of miR-199a-3p protects against this alteration. (B) Quantitative analysis of bone microarchitecture parameters indicates that GCs cause decreases in BV/TV, Tb.N, and Tb.Th, along with an increase in Tb.Sp, while simultaneous silencing of miR-199a-3p mitigates these changes (all *n* = 4). (C) H&E staining reveals that GCs cause trabecular bone sparsification, while simultaneous silencing of miR-199a-3p rescues this alteration. (D to F) IHC staining of ITGB8 (D), RUNX2 (E), and PPARγ (F) demonstrated that GCs suppress RUNX2 and ITGB8 expression while promoting PPARγ expression, and simultaneous miR-199a-3p silencing rescues these alterations. (G) Quantitative analysis of ITGB8-, RUNX2-, and PPARγ-positive area (all *n* = 4). (Data are presented as mean ± SD; one-way ANOVA was used; ns, not significant, *P < 0.05, **P < 0.01, ***P < 0.001, ****P < 0.0001.)

Micro-CT analysis revealed that MPS treatment disrupted the trabecular architecture of the femoral head, whereas antagomiR-199a-3p treatment markedly ameliorated these structural abnormalities (Fig. 9A). Bone morphometric analysis showed that the MPS group exhibited lower bone volume/total volume (BV/TV), trabecular number (Tb.N), and trabecular thickness (Tb.Th) than did the control group, along with higher trabecular separation (Tb.Sp) (Fig. 9B). Notably, compared with the MPS + NC group, the MPS + antagomiR group displayed a marked increase in BV/TV (18.4% versus 27.3%) and Tb.N (2.17 versus 2.95/mm), indicating effective reconstruction of trabecular structure.

H&E staining further proved that miR-199a-3p silencing can protect bone architecture, as the MPS + antagomiR group showed more regularly arranged trabeculae and fewer empty lacunae compared with the MPS and MPS + NC groups (Fig. 9C). In addition, IHC analysis demonstrated that antagomiR-199a-3p treatment restored ITGB8 and RUNX2 expression while attenuating PPARγ up-regulation (Fig. 9D to G).

**Fig. 10.**
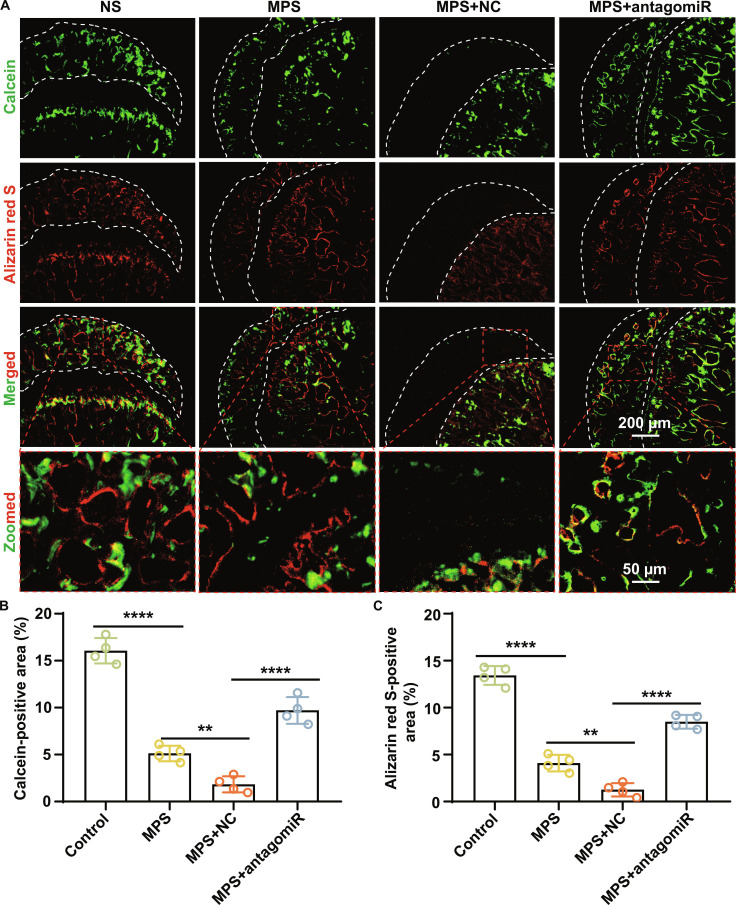
Silencing miR-199a-3p promotes new bone formation in GC-induced SONFH rats. (A) Representative fluorescent labeling of bone formation in rat femoral heads. Calcein (green, administered at week 2) and ARS (red, administered at week 4) were used for sequential labeling. Results indicate that GC treatment inhibits osteogenesis, manifested by reduced fluorescence areas of calcein (week 2) and ARS (week 4). Conversely, simultaneous miR-199a-3p silencing promotes new bone formation, evidenced by increased fluorescence areas of calcein and ARS. (B and C) Quantitative analysis of calcein-positive area (B) and ARS-positive area (C) (both *n* = 4). (Data are presented as mean ± SD; one-way ANOVA was used; **P < 0.01, ****P < 0.0001.)

Histomorphometric analysis, based on double-fluorochrome labeling, revealed continuous bands of newly formed bone in the control group (Fig. 10). In contrast, fluorescence intensity and labeled surface were markedly reduced in the MPS and MPS + NC groups. AntagomiR-199a-3p treatment substantially restored both fluorescence intensity and labeled area, indicating a significant improvement in bone formation rate (both *P* < 0.0001; Fig. 10B and C).

Collectively, miR-199a-3p silencing effectively ameliorates the disrupted bone microenvironment and promotes bone repair in SONFH, further supporting its feasibility as a potential therapeutic strategy.

## Discussion

GCs are now widely used; however, their long-term administration can induce SONFH, which is a debilitating complication that severely compromises hip function and for which effective early-stage interventions remain limited [24,25]. One of the earliest pathological events in SONFH is the aberrant OGD–AGD in BMSCs [5,6]. Consistent with prior studies linking early-stage SONFH to BMSC fate drift, nevertheless, the upstream drivers and actionable molecular regulators underlying this lineage imbalance have yet to be elucidated. In this study, by integrating miRNA microarray data related to the AGD of MSCs with exosomal miRNA sequencing data from SONFH patients, we systematically identified consistently up-regulated candidates and pinpointed miR-199a-3p as a key molecule. Notably, miR-199a-3p emerged as a prioritized candidate with prominent and reproducible up-regulation across patient exosomes, clinical tissues, and GC-exposed cellular models. Through a combination of clinical tissue validation, cellular functional assays, and in vivo intervention experiments, we delineated the pathogenic role of miR-199a-3p in GC-induced differentiation imbalance. Mechanistically, miR-199a-3p dampened ITGB8-dependent signaling and thereby impaired the osteogenic FAK–ERK–RUNX2 cascade, biasing BMSCs away from OGD to AGD. Furthermore, both in vitro functional rescue experiments and local in vivo administration confirmed the value of miR-199a-3p as a promising therapeutic target. Together, these findings provide an integrated framework that links exosomal miRNA dysregulation to a druggable membrane-to-nucleus signaling axis in SONFH.

Recent studies have demonstrated that aberrant epigenetic regulation, including dysregulated miRNAs, contributes to the pathogenesis of SONFH by disrupting the balance between OGD and AGD of BMSCs [12,26]. However, many reported miRNA candidates are supported primarily by single-layer evidence (e.g., a single model or assay), and their linkage to patient-derived exosomal cues and drug-exposure contexts remains incompletely defined. Building on this framework, our study nominates miR-199a-3p through a patient exosome–anchored, adipogenesis-informed prioritization strategy and supports it as an upstream contributor in GC-relevant settings. Across independent datasets and disease-relevant models, miR-199a-3p shows reproducible elevation, consistent with GC-associated homeostatic perturbations. Functionally, miR-199a-3p behaves as a bidirectional fate regulator whose net effect under GC exposure is to shift BMSC commitment away from osteogenesis and toward adipogenesis, an effect that remains reversible upon miR-199a-3p inhibition. Previous work has reported that miR-199a-3p can inhibit OGD by targeting Kdm3a and suppressing the ERK2/KLF2 pathway [14] or promote AGD via repression of KDM6A and the Wnt/β-catenin pathway [27]. In adipocytes, miR-199a-3p enhances lipid accumulation by regulating hypoxia-inducible factor-1α (HIF-1α) [28,29], supporting its sustained activity in AGD. Although these studies collectively suggest a role for miR-199a-3p in differentiation, they largely lack systematic validation in the context of GC-induced pathology. By combining gain- and loss-of-function approaches with GC exposure models, our study provides the systematic functional evidence that miR-199a-3p drives GC-induced OGD–AGD imbalance, highlighting its central pathogenic role in abnormal differentiation.

We further elucidated the mechanism by which miR-199a-3p regulates the OGD–AGD of BMSCs through direct targeting of ITGB8. Given the established role of integrin–FAK signaling in mechanotransduction and osteogenic commitment, ITGB8 was confirmed as a direct target of miR-199a-3p by dual-luciferase and protein analyses. In both clinical tissues and GC-induced models, miR-199a-3p and ITGB8 showed inverse expression patterns, suggesting coordinated dysregulation under pathological conditions. Functionally, ITGB8 repression attenuates p-FAK/p-ERK signaling and weakens RUNX2-driven osteogenesis while favoring AGD. Importantly, rescue experiments position ITGB8 as a key effector of miR-199a-3p-mediated fate drift. The FAK–ERK–RUNX2 pathway is a well-recognized signaling axis by which BMSCs sense mechanical cues and maintain osteogenic lineage commitment [30,31]. Its activity has also been implicated in the negative regulation of AGD by suppressing PPARγ [32,33]. As a transmembrane integrin receptor, ITGB8 forms heterodimers with its partner subunit ITGAV, which has been reported to regulate the OGD–AGD balance of BMSCs [34,35], suggesting a broader role of this integrin family in fate specification. Building on this conceptual framework, our study provides experimental evidence validating ITGB8 as a regulator of the OGD–AGD fate balance in BMSCs and placing it within a miRNA-mediated regulatory network. Collectively, we delineate a miR-199a-3p–ITGB8–FAK–ERK–RUNX2 signaling axis that orchestrates OGD–AGD imbalance, thereby establishing a pathogenic mechanistic loop that spans epigenetic regulation, membrane receptor signaling, and nuclear transcriptional responses.

Based on the mechanistic identification of the miR-199a-3p–ITGB8–FAK–ERK regulatory axis, we further evaluated it as an intervention target. In GC-treated BMSCs, miR-199a-3p silencing effectively relieved ITGB8 repression, reactivated the FAK–ERK–RUNX2 pathway, and markedly corrected GC-induced suppression of OGD and enhancement of AGD. More importantly, in a rat SONFH model, local administration of antagomiR-199a-3p robustly increased ITGB8 expression, restored the balance between bone and fat differentiation, improved trabecular architecture, and increased bone mass, thereby validating its therapeutic efficacy in bone repair. These findings align with emerging efforts to therapeutically modulate miRNA programs in skeletal disorders while providing a mechanistically anchored and locally deliverable target in SONFH. Considering previous reports of sustained miR-199a-3p up-regulation in osteoporosis and other bone metabolic disorders [14], together with our findings, miR-199a-3p may represent a shared regulatory node of OGD–AGD fate imbalance and therefore represents a potentially targetable molecule across multiple bone disease contexts. In addition, the local antagomir delivery strategy employed in this study provides a means to precisely modulate stem cell fate within the bone microenvironment while potentially minimizing systemic exposure, offering a feasible route for miRNA-based therapeutics in skeletal disorders. Taken together, our data support miR-199a-3p as a mechanistically grounded, reversibly targetable molecule that holds promise as a key effector for miRNA-guided intervention in SONFH.

Despite these findings, several limitations must be acknowledged. First, the size of clinical samples was small (particularly for exosome analysis), and the study design was predominantly cross-sectional. Thus, the dynamic relationship between miR-199a-3p levels and SONFH staging or prognosis could not be fully assessed. Second, although we validated this regulatory axis in rat BMSCs (rBMSCs), MC3T3-E1 cells, and a rat SONFH model, additional validation in human primary BMSCs and in models more closely resembling clinical SONFH (e.g., bone organoids constructed from SONFH patient-derived BMSCs) would enhance its translational significance. Third, given the multi-target nature of miRNAs, ITGB8, while a key effector molecule, may not be the sole mediator. Other target interactions and cross-regulatory pathways (such as KDM6A and CD44, as we identified) may participate in the regulation. Finally, while locally targeted delivery of antagomiR demonstrated efficacy, comprehensive evaluation of long-term safety, dose optimization, tissue distribution, and potential off-target effects remains necessary.

Future research may expand upon this work in the following directions: (a) validate circulating or exosomal miR-199a-3p as a biomarker for early risk assessment or monitoring of anti-resorptive/pro-osteogenic therapies in large, phenotypically defined cohorts stratified by imaging, e.g., FICAT staging for femoral head necrosis, and follow-up. (b) Mechanistic studies should elucidate how GC exposure up-regulates miR-199a-3p (e.g., via GC receptor-dependent transcriptional regulation or epigenetic remodeling). (c) Context-dependent regulation of the miR-199a-3p–ITGB8 axis can be revealed through cell-type-specific and microenvironmental analyses, including ITGAV–ITGB8 pairing effects, extracellular matrix signaling, and mechanical signaling influences. (d) Finally, optimizing bone-targeting local delivery platforms and conducting rigorous safety/toxicology studies will be critical steps for achieving the clinical translation of SONFH miRNA-targeted therapy.

## Conclusion

In summary, this study first reveals a key epigenetic pathogenic axis in SONFH: GC induces the up-regulation of miR-199a-3p, which targets and suppresses ITGB8, thereby inhibiting the FAK–ERK–RUNX2 osteogenic signaling cascade. This change drives an imbalance in OGD–AGD of BMSCs and promotes SONFH progression. Through in vivo and in vitro validation, we further demonstrate that silencing miR-199a-3p restores ITGB8 expression, reactivates this signaling cascade, and improves bone microarchitecture, suggesting its potential therapeutic value. Future research will focus on developing specific local delivery strategies for miR-199a-3p, systematically evaluating its long-term efficacy and safety, and validating its translational potential as a biomarker for diagnostic stratification and therapeutic monitoring across large-scale cohorts at different disease stages.

## Materials and Methods

### Clinical samples and processing of bone tissues

Animal experiments were approved by the Ethics Committee of the First Affiliated Hospital of Chongqing Medical University (approval no. 2019-343). A total of 22 subjects were enrolled, including 11 patients diagnosed with SONFH and 11 patients with FNF serving as controls, all of whom underwent hip arthroplasty at the same hospital. Diagnoses of SONFH and FNF were based on combined radiographic (x-ray and CT) findings and confirmed by postoperative histopathological evaluation.

During surgery, femoral head specimens were divided into 2 parts: one for molecular analyses and the other for histological processing. Fixed samples were decalcified, paraffin-embedded, and sectioned for subsequent staining, including H&E staining and IHC staining of ITGB8, RUNX2, and PPARγ.

### IF staining

Cells were fixed, permeabilized, and blocked, then incubated overnight at 4 °C with primary antibodies. Alexa Fluor 488- or 647-conjugated secondary antibodies were applied for 2 h. Phalloidin–Alexa Fluor 555 or Alexa Fluor 488 and 4′,6-diamidino-2-phenylindole (DAPI) were used to stain F-actin and nuclei, respectively. They were observed and photographed, and the images were quantified using ImageJ.

### Dual-luciferase reporter assay

WT- or MT-ITGB8 3′UTR fragments were cloned into the GP-miRGLO luciferase reporter vector (GenePharma, China) and sequence-verified. HEK-293T cells were cotransfected with the reporter plasmids and agomiR-199a-3p or NC. Luciferase activity was measured using the Promega Dual-Luciferase Reporter Assay System.

### Bioinformatic analyses

To identify key miRNAs associated with AGD and SONFH, the miRNA expression dataset GSE59684, derived from AGD of human MSCs (hMSCs), was obtained from the Gene Expression Omnibus (GEO) database. GSE59684 is a noncoding RNA (miRNA) microarray dataset generated on the miRCURY LNA microRNA Array, 6th generation platform (GPL17382; Exiqon). Samples at days 0 and 13 of adipogenic induction were selected for comparison. GEO2R was used to perform the online analysis of GSE59684, following the default workflow for sample grouping, quality assessment, and miRNA expression matrix preparation for subsequent differential expression analysis. Additionally, data normalization was automatically performed by GEO2R in the background, using default settings based on the limma framework, including log_2_ transformation and platform-adaptive normalization. The screening criteria were set as |log₂FC| > 0.585 and adjusted *P* value (adj. *P*) < 0.05. Next, we incorporated our previously reported miRNA-sequencing data of exosomes from SONFH patients (16). A Venn diagram-based intersection analysis was then performed between exosomal candidate miRNAs and differentially expressed miRNAs from GSE59684, yielding a subset of putative key regulatory miRNAs associated with both AGD and SONFH progression for subsequent functional studies.

Furthermore, to identify candidate target genes functionally related to miR-199a-3p, the mRNA expression dataset GSE36923, which profiles gene expression during AGD of hMSCs, was retrieved from the GEO database. GSE36923 is a gene expression microarray dataset generated on the Affymetrix Human Genome U133 Plus 2.0 Array platform (GPL570). GEO2R was used to perform the online analysis of GSE36923, following the default workflow for sample grouping, quality assessment, and preparation of the probe-annotated gene expression matrix for subsequent differential expression analysis. Data normalization for GSE36923 was likewise automatically performed by GEO2R in the background, under default settings based on the limma framework, including log_2_ transformation and platform-adaptive normalization procedures. DEGs were identified with screening thresholds set at *P* < 0.05 and |log₂FC| > 1. Predicted targets of miR-199a-3p were first obtained from the TargetScan 8.0 database, and conserved target genes were defined as the intersection of predicted targets in humans, rats, and mice. These conserved targets were subsequently imported into the STRING database to construct a protein–protein interaction (PPI) network. The Maximal Clique Centrality method of the CytoHubba plugin in Cytoscape software was then applied to identify the top 15 hub genes. Finally, the DEGs from GSE36923 were intersected with these top 15 hub genes to obtain candidate genes that were both altered during AGD and located at central positions within the miR-199a-3p target network, which were selected for further experimental validation.

Methods for RNA extraction from bone tissue, exosomes, and cells, RT-qPCR, cell culture and transfection, protein extraction and WB, OGD and staining, AGD and Oil Red O (ORO) staining, animal experiments, and statistical analysis are detailed in Sections S2.1 to S2.8.

Primer sequences for RT-qPCR are provided in Table 1.

**Table 1.** Primer sequences in this study

Gene	Forward (5′ to 3′)	Reverse (5′ to 3′)
miR-21-5p	CGGCGTAGCTTATCAGACTGATGTTGA	B661601 (Sangon Biotech)
miR-27b-3p	CGGTTCACAGTGGCTAAGTTCTGC	B661601 (Sangon Biotech)
miR-199a-3p	GGAACCCGTAGATCCGAACTTGTG	B661601 (Sangon Biotech)
miR-125b-1-3p	ATTACGGGTTAGGCTCTTGGGAG	B661601 (Sangon Biotech)
miR-196a-5p	CGGTAGGTAGTTTCATGTTGTTGGG	B661601 (Sangon Biotech)
miR-183-5p	CCGTATGGCACTGGTAGAATTCACT	B661601 (Sangon Biotech)
miR- miR-107	AGCAGCATTGTACAGGGCTATCA	B661601 (Sangon Biotech)
U6	B661602 (Sangon Biotech)	B661601 (Sangon Biotech)
Rat ITGB8	GCTGGGCATGGAGAGTGTGAAG	GTGCTGGGCTGCTGCTGAAG
Rat CD44	TGCCGTTACGCAGGTGTATT	CATGGTGGGCAAGGTGGTAT
Rat KDM6A	CCACCCTGCCTAGCAATTCA	GGCAGTGCTGTTAGGTGTCT
Rat PDGFRA	CAGGCCTTTCTGGTCCTCAG	TCCCCGAAGCATCTCAGAGA
Rat RUNX2	TCCGCCACCACTCACTACCAC	GGAACTGATAGGACGCTGACGAAG
Rat OCN	GGACCCTCTCTCTGCTCACTCTG	ACCTTACTGCCCTCCTGCTTGG
Rat FABP4	AACTGGGCGTGGAATTCGAT	TGGTCGACTTTCCATCCCAC
Rat PPARγ	CCATCGAGGACATCCAAGACAACC	GTGCTCTGTGACAATCTGCCTGAG
Mouse RUNX2	CGGGAATGATGAGAACTACTC	GTGAAACTCTTGCCTCGTCCG
Mouse OCN	CTGACCTCACAGATCCCAAGC	TGGTCTGATAGCTCGTCACAAG
Mouse FABP4	TCACCGCAGACGACAGGAAG	CACCACCAGCTTGTCACCA
Mouse PPARγ	GTACTGTCGGTTTCAGAAGTGCC	ATCTCCGCCAACAGCTTCTCCT
Rat GAPDH	GACATGCCGCCTGGAGAAAC	AGCCCAGGATGCCCTTTAGT
Mouse GAPDH	GGTTGTCTCCTGCGACTTCA	TGGTCCAGGGTTTCTTACTCC

## Data Availability

The miRNA microarray data (GSE59684) and mRNA sequencing data (GSE36923) are available in the GEO database (https://www.ncbi.nlm.nih.gov/geo/). Experimental data will be made available on request.
