# Supplementary material for: miR-199a-3p Promotes Adipogenic Differentiation to Aggravate Steroid-Induced Osteonecrosis of Femoral Head via the ITGB8/FAK–ERK/RUNX2 Pathway
Source: Research (Wash D C). 2026 Mar 23;9:1186. doi: 10.34133/research.1186 (PMC13006736; doi:10.34133/research.1186)
Supplement: Supplementary 1 — Supplementary Text Tables S1 to S3 Figs. S1 to S10 [file research.1186.f1.zip › Supplementary materials.docx]

Supplementary Figures for

**miR-199a-3p Promotes Adipogenic Differentiation to Aggravate Steroid-Induced Osteonecrosis of Femoral Head via the ITGB8/FAK-ERK/RUNX2 Pathway**

This file includes Supplementary Methods 1.1-1.8 and Fig. S1-S4.

**1. Abbreviations**

3′-UTR, 3′ Untranslated region
ALP, Alkaline phosphatase
ARS, Alizarin Red S
BMSCs, Bone marrow mesenchymal stem cells
BV/TV, Bone volume/total volume
cDNA, Complementary DNA
Col I, Collagen Type I
DEGs, Differentially expressed genes
ERK, Extracellular signal-regulated kinase
FAK, Focal adhesion kinase
FNF, Femoral neck fracture
GC, Glucocorticoid
GEO, Gene Expression Omnibus
hMSCs, Human mesenchymal stem cells
H&E, Hematoxylin and eosin

IF, Immunofluorescence
IHC, Immunohistochemistry
ITGB8, Integrin β8
MPS, Methylprednisolone
MT-ITGB8, Mutant ITGB8 plasmid
PPI, Protein–protein interaction
SD, Standard deviation
siITGB8, Small interfering RNA targeting ITGB8
SONFH, Steroid-induced osteonecrosis of the femoral head
Tb.N, Trabecular number
Tb.Sp, Trabecular separation
Tb.Th, Trabecular thickness
WB, Western blotting
WT-ITGB8, Wild-type ITGB8 plasmid

**2. Supplementary Methods**

**2.1 RNA Extraction from Bone Tissue, Exosomes, and Cells**

Exosome isolation was performed as previously described by our group (1). Briefly, bone specimens were initially fragmented using rongeurs and subsequently ground into fine powder under liquid nitrogen. The tissue homogenate was prepared in DEPC-treated PBS and centrifuged sequentially at 300 g for 10 minutes, 1,500 g for 10 minutes, and 10,000 g for 30 minutes. The resulting supernatant was ultracentrifuged at 100,000 g for 2 × 70 min to obtain exosomes. Total miRNA was extracted from cells, exosomes, and liquid nitrogen-processed bone tissues using the Biospin miRNA Extraction Kit (BioFlux, China) according to its guidelines. A Simply P Total RNA Extraction Kit (BioFlux, China) was used to isolate total RNA. RNA concentrations were measured.

**2.2 RT-qPCR**

Complementary DNA (cDNA) synthesis of miRNA was performed using a miRNA First-Strand cDNA Synthesis Kit (poly(A) tailing method; Sangon Biotech, China). RT-qPCR reaction steps are as follows: 95°C for 10 minutes, followed by 40 cycles of 95°C for 10 seconds, 58°C for 20 seconds, and 72°C for 10 seconds.

For mRNA synthesis, the RNA was first reverse-transcribed into cDNA using a kit (Takara, Japan). The resulting cDNA was then diluted 1:10 for subsequent assays. RT-qPCR reaction steps are as follows: 95°C for 5 minutes, followed by 40 cycles of 95°C for 10 seconds and 60°C for 30 seconds.

U6 served as the internal control (IC) for miRNA, while GAPDH was used as the IC for mRNA. The relative expression of RNA was calculated using the ^2-ΔΔ^Ct method (2). Primer sequences are provided in **Table 1**.

**2.3 Cell Culture and Transfection**

Rat BMSCs (rBMSCs; Cyagen, China), human embryonic kidney HEK-293T cells, and MC3T3-E1 pre-osteoblasts (National Cell Bank, Shanghai) were cultured in high-glucose DMEM (HyClone, UK) supplemented with 10% fetal bovine serum (Gibco, UK) and 1% penicillin-streptomycin at 37°C in 5% CO₂. Dexamethasone (DEX, 20 µM) was used to induce glucocorticoid stimulation (3).

When cells reached approximately 50% confluence, they were treated with dexamethasone and/or transfected with Endofectin™-MAX (GeneCopoeia, USA). The following oligonucleotides and plasmids (GenePharma, China) were used: negative control (NC), agomiR-199a-3p (100 nM), antagomiR-199a-3p (200 nM), siRNA targeting ITGB8 (siITGB8, 200 nM), wild-type ITGB8 plasmid (WT-ITGB8), and mutant-type ITGB8 plasmid (MT-ITGB8).

**2.4 Protein Extraction and Western Blotting (WB)**

Total protein from cells was extracted by RIPA (Beyotime, China) supplemented with phosphatase inhibitors (Abcam, U.S.A). Protein concentrations were determined with the BCA assay (Beyotime, China). Equal amounts of protein (30 μg) were separated via 10% SDS-PAGE and transferred to PVDF membranes (Millipore, U.S.A). After blocking, membranes were incubated overnight at 4°C with primary antibodies against β-actin, RUNX2, FAK, PPARγ, ERK1/2, p-ERK1/2, p-FAK, and ITGB8. HRP-conjugated secondary antibodies were applied for 1 h at room temperature, and signals were detected using an ECL kit (ZenBio, China). The information about the antibodies used in this study is shown below.

| Antibody name | Supplier | Code | Dilution |
| --- | --- | --- | --- |
| RUNX2 | Proteintech | 20700-1-AP | 1:1000 for WB, 1:150 for IHC |
| PPARγ | Proteintech | 16643-1-AP | 1:1000 for WB, 1:150 for IHC |
| ITGB8 | Zenbio | 508433 | 1:1000 for WB, 1:150 for IHC |
| ITGB8 | Abcam | ab317020 | 1:400 for IF staining |
| Collagen type I | CST |  | 1:200 for IF staining |
| FABP4 | Proteintech | 12802-1-AP | 1:200 for IF staining |
| p-FAK | CST | 3283 | 1:1000 for WB |
| FAK | Wanlei Bio | WL03350 | 1:1000 for WB |
| β-actin | Affinity | AF7018 | 1:5000 for WB |
| p-ERK1/2 | CST | 4370 | 1:1000 for WB |
| ERK1/2 | CST | 4695 | 1:1000 for WB |
| Goat-anti Rabbit IgG | Biosharp | BL003A | 1:8000 for WB |
| Goat-anti Mouse IgG | Biosharp | BL021A | 1:8000 for WB |
| Actin-tracker 555 | Beyotime | C2201S | 1:400 for IF staining |
| Actin-tracker 488 | Beyotime | C2203S | 1:400 for IF staining |
| AF488 Fluorescent Secondary Antibody | Beyotime | A0423 | 1:200 for IF staining |
| AF647 Fluorescent Secondary Antibody | Beyotime | A0468 | 1:200 for IF staining |

**2.5 Osteogenic Differentiation (OGD) and Staining**

Cells were cultured in OGD medium (Cyagen, China) once reaching 80% confluence. After 1 week of induction, alkaline phosphatase (ALP) staining was performed using a kit (Beyotime, China). After 3 weeks, Alizarin Red S (ARS) staining (Solarbio, China) was conducted. Calcium nodules were extracted using 10% cetylpyridinium chloride (Sangon Biotech, China) and measured at OD 562 nm to quantify mineralization (4).

**2.6 Adipogenic Differentiation (AGD) and Oil Red O (ORO) Staining**

Cells were induced with AGD medium (Cyagen, China) once fully confluent. After 3 weeks, lipid droplets were stained using an ORO Staining Kit (Solarbio, China). Lipid accumulation was quantified by isopropanol extraction, followed by absorbance measurement at 518 nm (5).

**2.7 Animal Experiments**

Animal experiments were approved by the Ethics Committee of the First Affiliated Hospital of Chongqing Medical University (Approval No. 2020-520) and conducted in accordance with NIH guidelines. Sixteen female Sprague-Dawley rats (8 weeks old) were randomly assigned to four groups (n = 4 each): Control, Methylprednisolone (MPS), MPS+NC, and MPS+antagomiR-199a-3p. MPS was administered intramuscularly, while NC and antagomiR-199a-3p were delivered via periosteal injection. Calcein (10 mg/kg) and Alizarin Red S (30 mg/kg) were injected at weeks 2 and 4 for fluorescent labelling (6). Rats were euthanized at week 6, and femoral heads were collected for micro-CT examination and histological analyses.

Micro-CT scanning was performed using a Bruker Skyscan 1174 system. Three-dimensional reconstruction and quantitative analyses were conducted to evaluate trabecular parameters, including BV/TV, Tb.N, Tb.Th, and Tb.Sp. After fixation and decalcification, H&E staining and immunohistochemistry for ITGB8, RUNX2, and PPARγ were performed.

For fluorochrome labelling, the femoral heads were sectioned at a thickness of 10 μm. Confocal microscopy (Leica) was used to capture images with the following excitation/emission settings: Alizarin Red (543 nm/580-670 nm) and calcein (488 nm/500-550 nm).

**2.8 Statistical Analysis**

All results are expressed as the mean ± standard deviation (SD). Statistical analyses were carried out using GraphPad Prism 8.0. Differences between the two groups were assessed with Student's *t*-test, while comparisons among multiple groups were evaluated using one-way ANOVA followed by Tukey's post hoc analysis. A *p*-value less than 0.05 was considered statistically significant.

**3. Supplementary Figures**

**
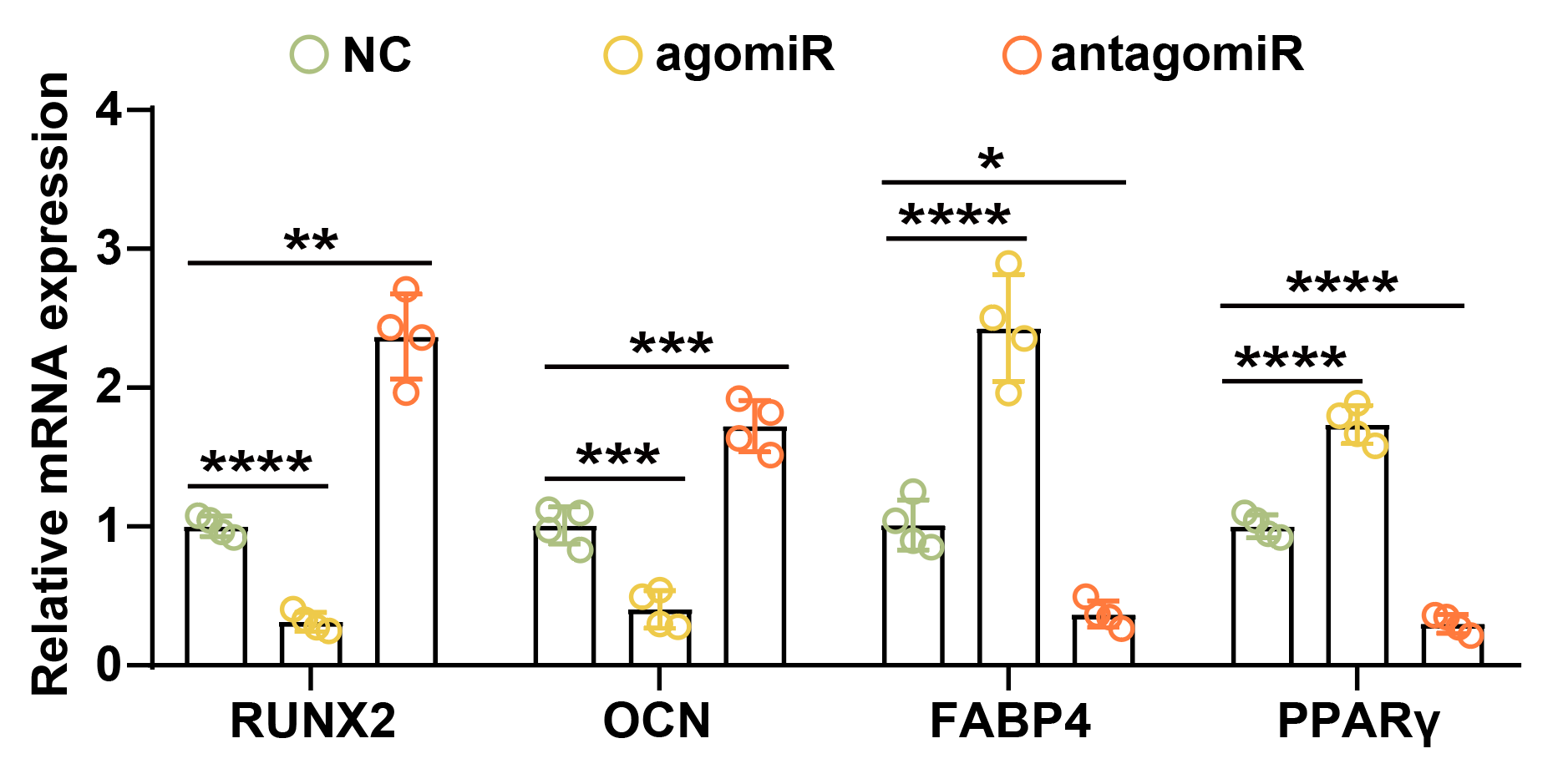
**

**Fig. S1.** RT-qPCR for the expression of RUNX2, OCN, FABP4, and PPARγ in rBMSCs transfected with NC, agomir-199a-3p, and antagomir-199a-3p, respectively (all n = 4). (Data are presented as mean ± SD, one-way ANOVA was used; ns means *p* > 0.05, ** means *p* < 0.01, *** means *p* < 0.001)

**
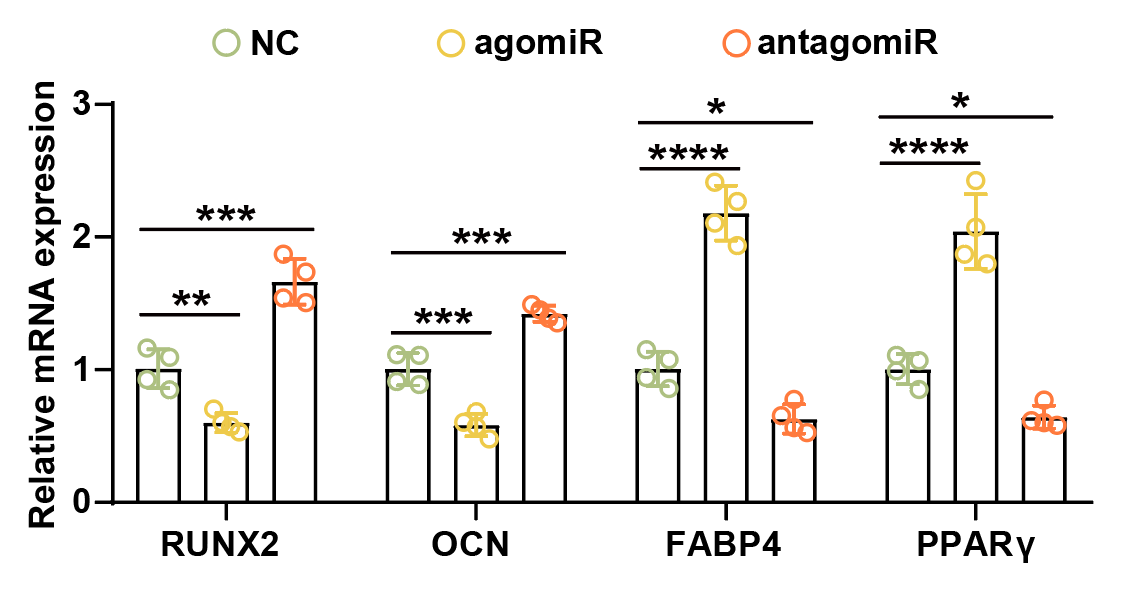
**

**Fig. S2.** RT-qPCR for the expression of RUNX2, OCN, FABP4, and PPARγ in MC3T3-E1 cells transfected with NC, agomir-199a-3p, and antagomir-199a-3p, respectively (all n = 4). (Data are presented as mean ± SD, one-way ANOVA was used; ns means *p* > 0.05, ** means *p* < 0.01, *** means *p* < 0.001)


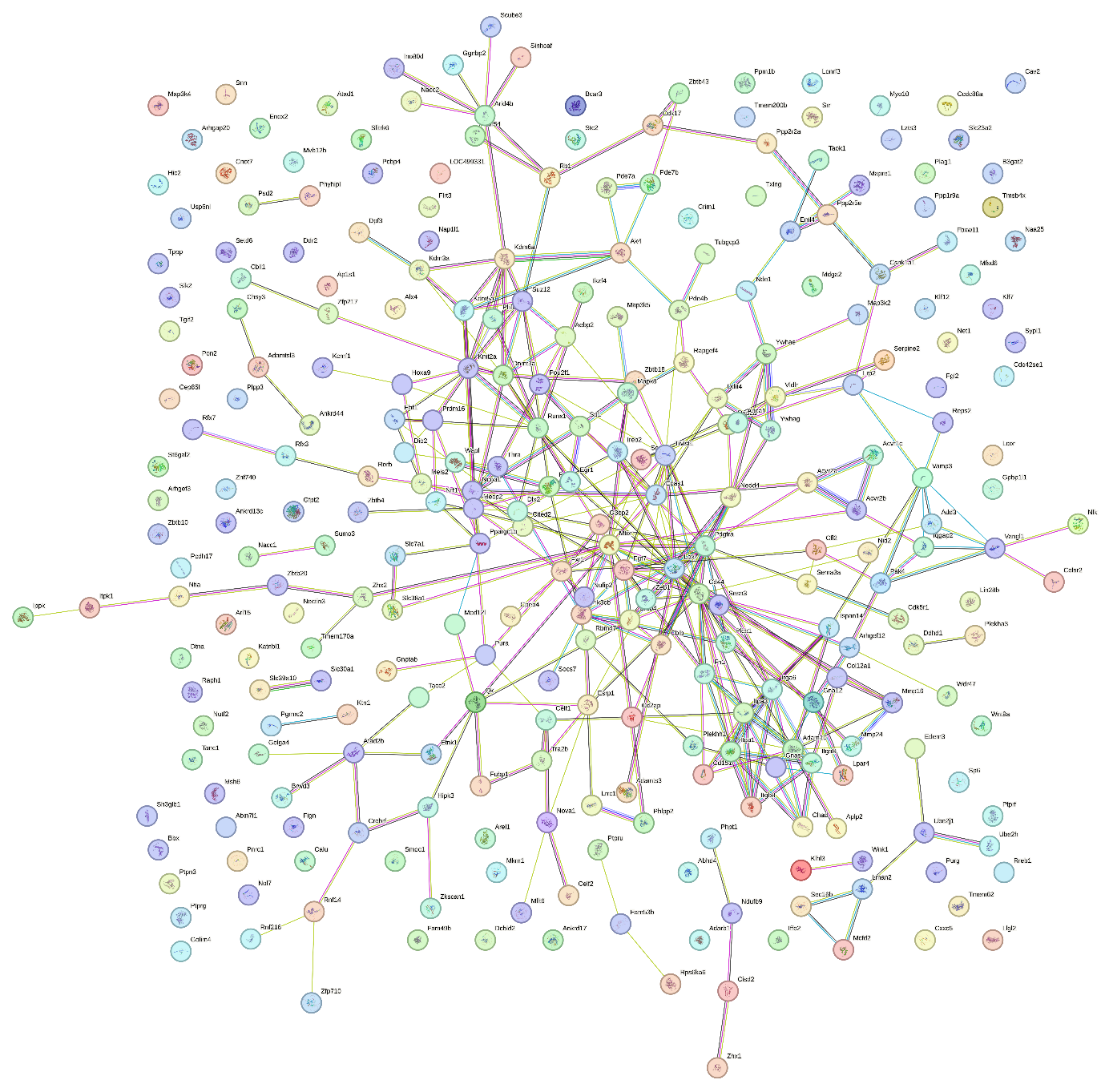


**Fig. S3.** PPI network of the 294 target genes for miR-199a-3p


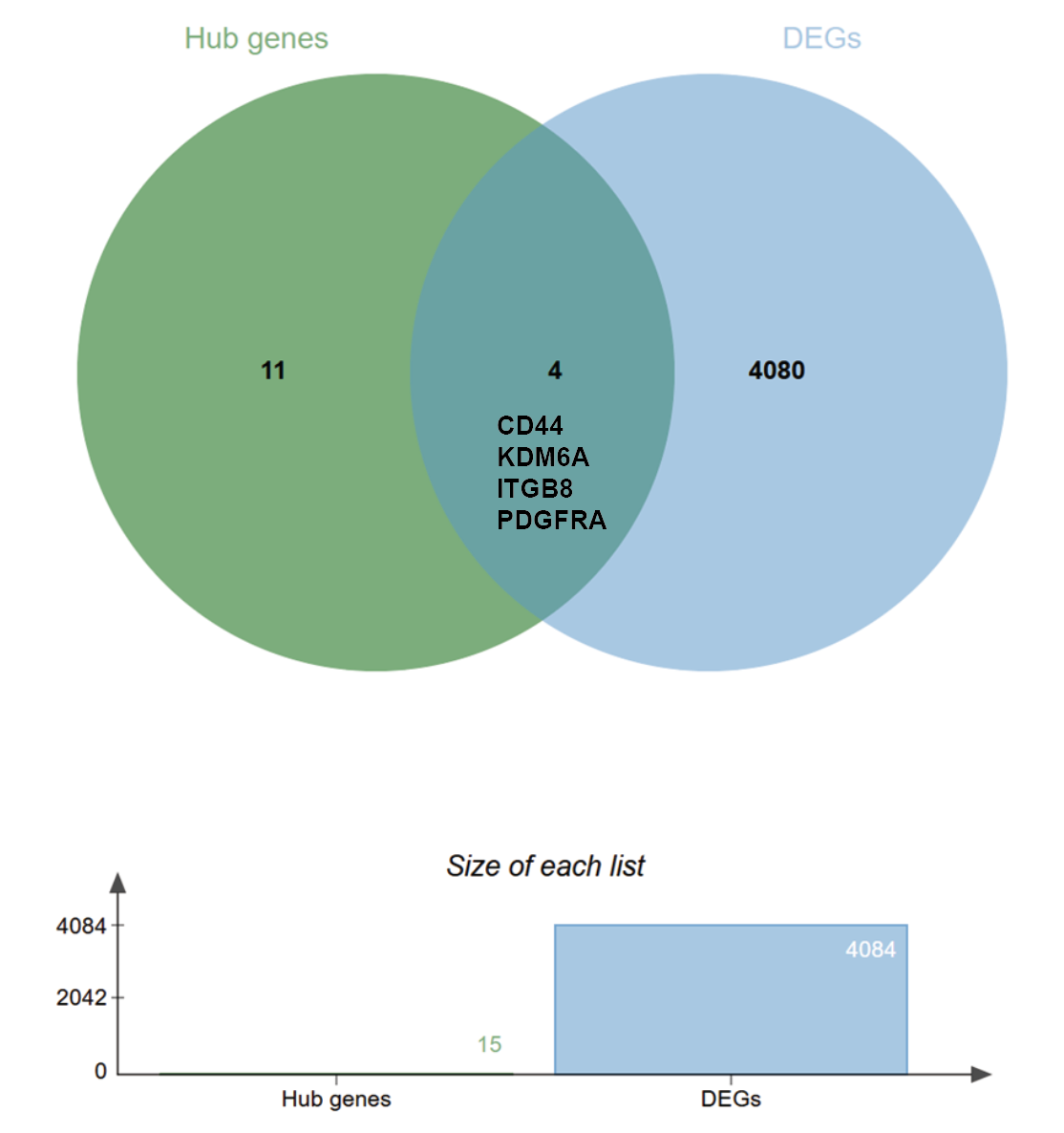


**Fig. S4.** Venn diagram illustrating hub genes among miR-199a-3p target genes, as well as DEGs during adipogenic differentiation. Four genes are identified: CD44, KDM6A, ITGB8, and PDGFRA.


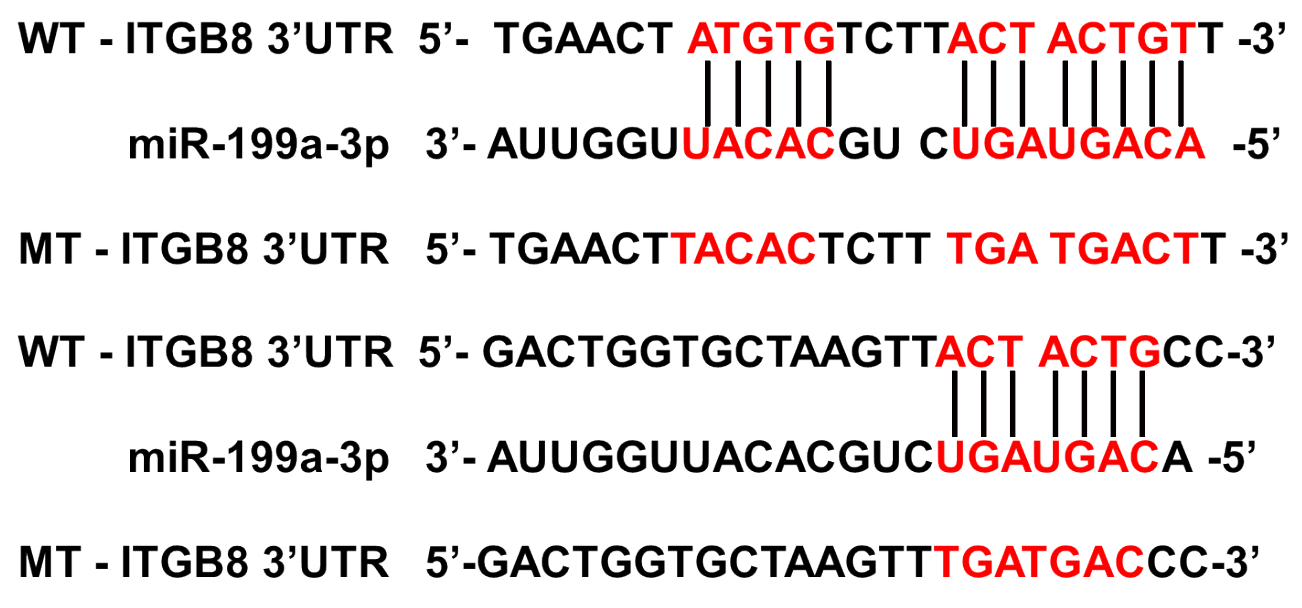


**Fig. S5.** Schematic of WT and MT ITGB8 3'UTR luciferase reporter constructs

**
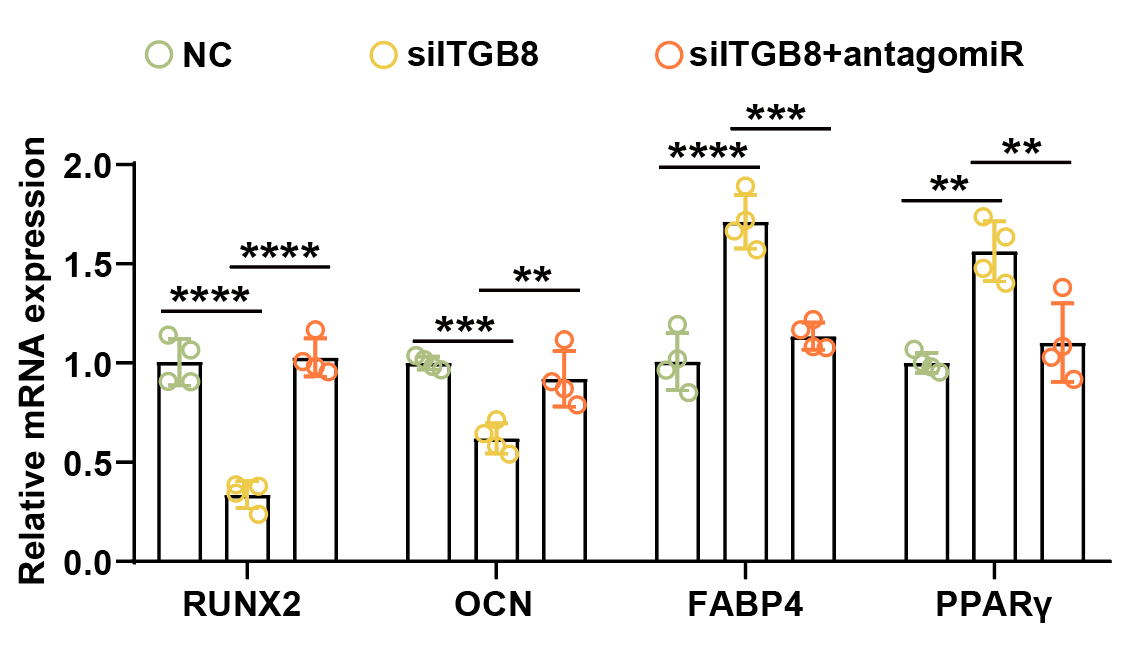
Fig. S6.** RT-qPCR for the expression of RUNX2, OCN, FABP4, and PPARγ in rBMSCs transfected with NC, siITB8, and siITB8+antagomir-199a-3p, respectively (all n = 4). (Data are presented as mean ± SD, one-way ANOVA was used; ** means *p* < 0.01, *** means *p* < 0.001, **** means *p* < 0.0001)

**
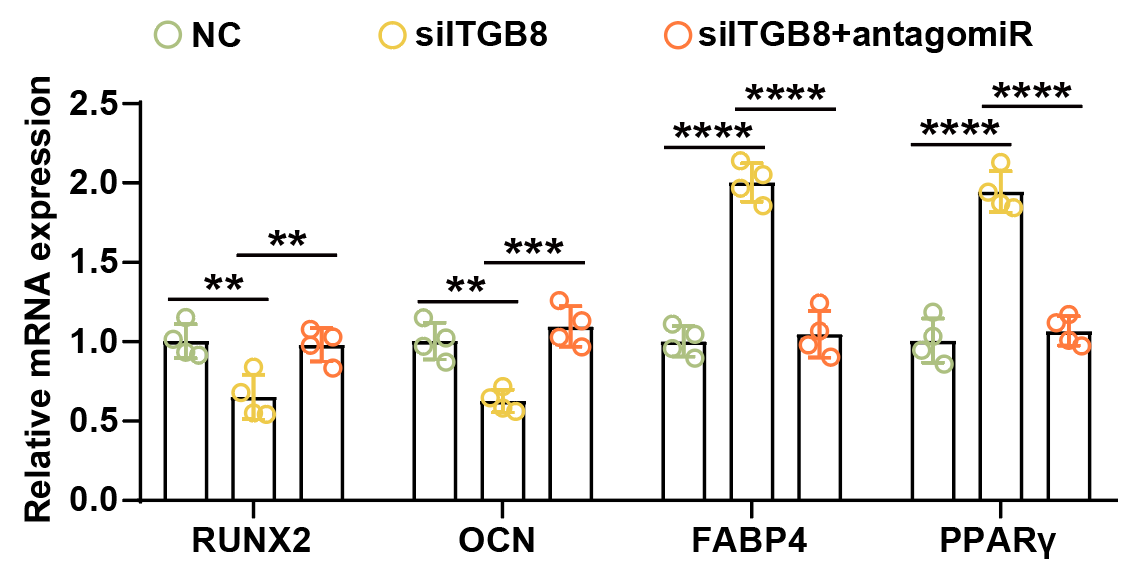
**

**Fig. S7.** RT-qPCR for the expression of RUNX2, OCN, FABP4, and PPARγ in MC3T3-E1 cells transfected with NC, siITB8, and siITB8+antagomir-199a-3p, respectively (all n = 4). (Data are presented as mean ± SD, one-way ANOVA was used; ** means *p* < 0.01, *** means *p* < 0.001, **** means *p* < 0.0001)


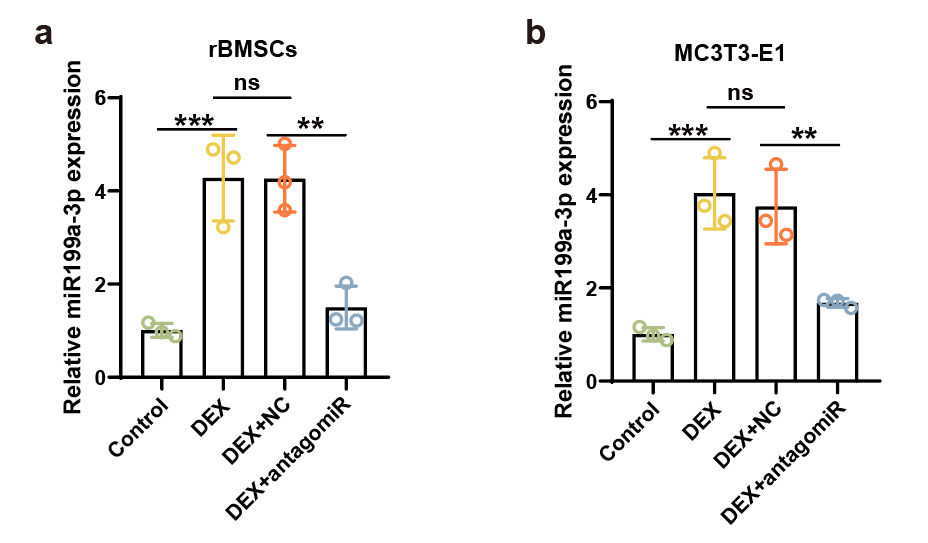


**Fig. S8.** RT-qPCR for miR-199a-3p expression (all n = 3). (Data are presented as mean ± SD, one-way ANOVA was used; ns means *p* > 0.05, ** means *p* < 0.01, *** means *p* < 0.001)

**
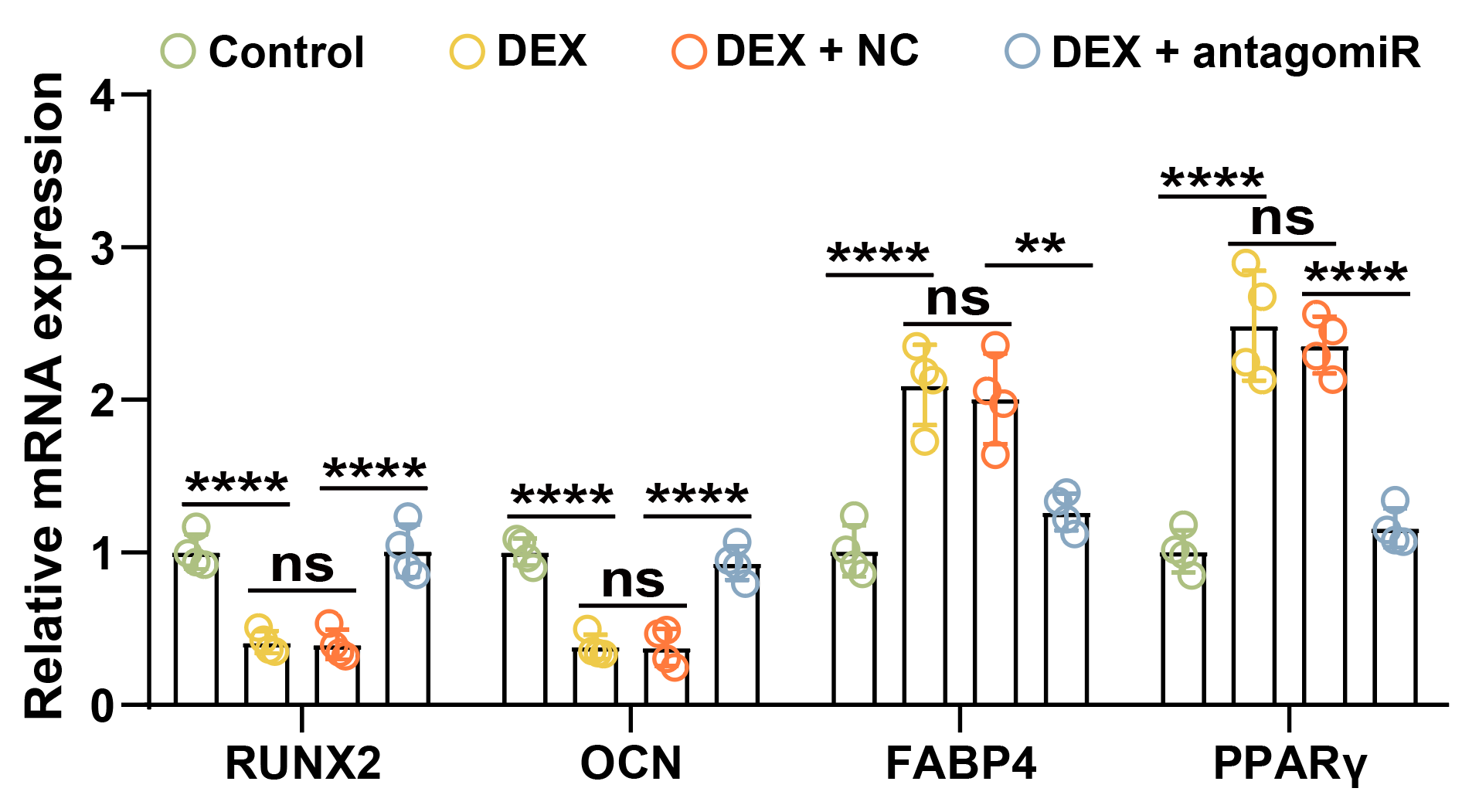
**

**Fig. S9.** RT-qPCR for the expression of RUNX2, OCN, FABP4, and PPARγ in MC3T3-E1 cells transfected with NC, siITB8, and siITB8+antagomir-199a-3p, respectively (all n = 4). (Data are presented as mean ± SD, one-way ANOVA was used; ns means *p* > 0.05, ** means *p* < 0.01, **** means *p* < 0.0001)


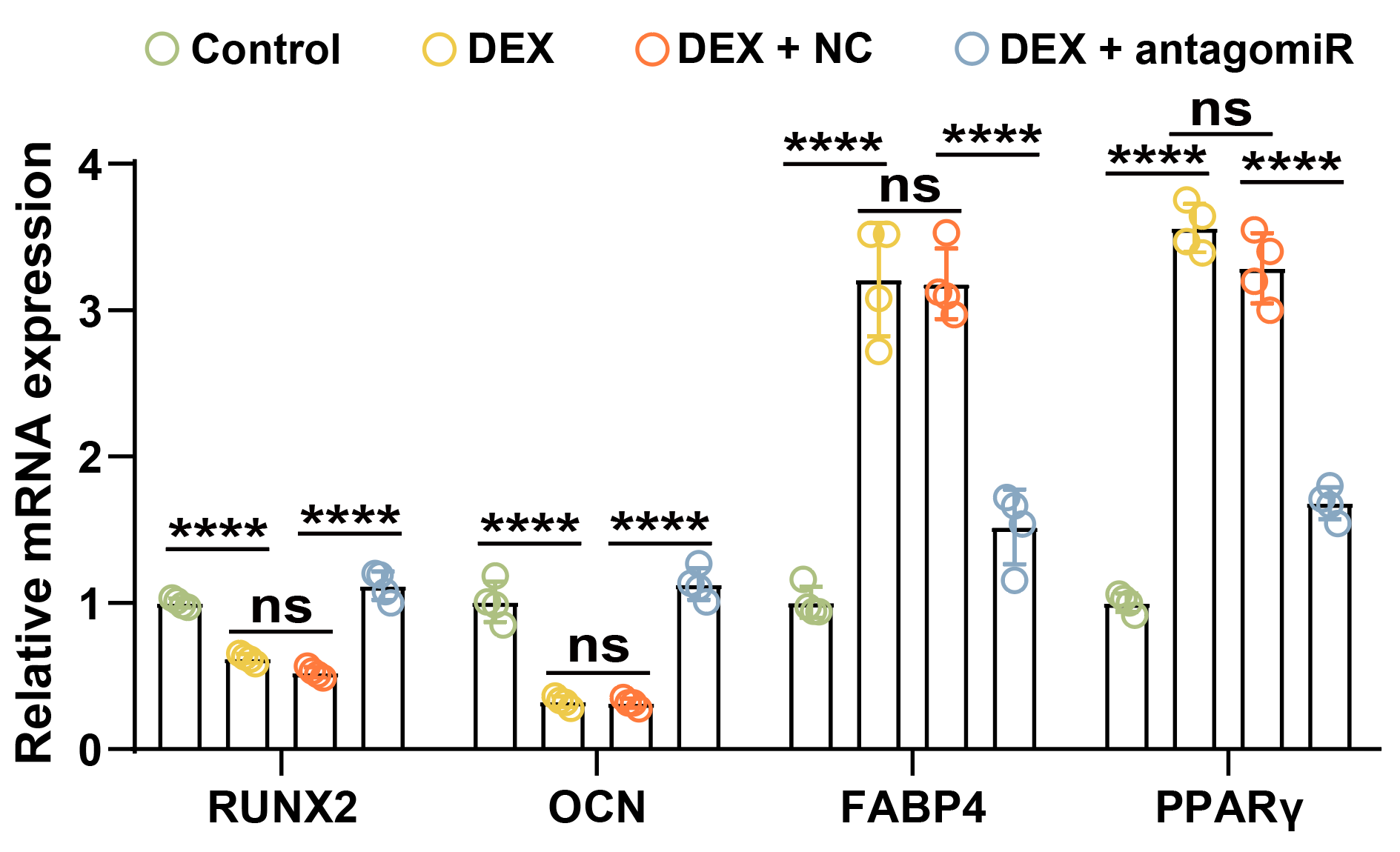
**Fig. S10.** RT-qPCR for the expression of RUNX2, OCN, FABP4, and PPARγ in MC3T3-E1 cells transfected with NC, siITB8, and siITB8+antagomir-199a-3p, respectively (all n = 4). (Data are presented as mean ± SD, one-way ANOVA was used; ns means *p* > 0.05, **** means *p* < 0.0001)

**References**

1. Yang W, Zhu W, Yang Y, Guo M, Qian H, Jiang W, et al. Exosomal miR-100-5p inhibits osteogenesis of hBMSCs and angiogenesis of HUVECs by suppressing the BMPR2/Smad1/5/9 signalling pathway. Stem cell research & therapy. 2021;12(1):390.

2. Livak KJ, Schmittgen TD. Analysis of relative gene expression data using real-time quantitative PCR and the 2− ΔΔCT method. methods. 2001;25(4):402-8.

3. Liu Q, Wu Y, Li S, Yoon S, Zhang J, Wang X, et al. Ursolic acid alleviates steroid-induced avascular necrosis of the femoral head in mouse by inhibiting apoptosis and rescuing osteogenic differentiation. Toxicology and Applied Pharmacology. 2023;475:116649.

4. Yang W, Ding T, Zhuang P, Chen Y, Zhang Y, Chen Z, et al. In situ construction of ossification micro-units for critical bone regeneration via sustained lifting of epigenetic suppression. Journal of Controlled Release. 2025:113978.

5. Martínez M, Blanco J, Rovira J, Kumar V, Domingo J, Schuhmacher M. Bisphenol A analogues (BPS and BPF) present a greater obesogenic capacity in 3T3-L1 cell line. Food and Chemical Toxicology. 2020;140:111298.

6. Yu H, Liu P, Zhu D, Yin J, Yang Q, Huang Y, et al. Chrysophanic acid shifts the differentiation tendency of BMSCs to prevent alcohol‐induced osteonecrosis of the femoral head. Cell Proliferation. 2020;53(8):e12871.
